# Temporal analyses reveal a pivotal role for sense and antisense enhancer RNAs in coordinate immunoglobulin lambda locus activation

**DOI:** 10.1093/nar/gkad741

**Published:** 2023-09-13

**Authors:** Zeqian Gao, Alastair L Smith, James N F Scott, Sarah L Bevington, Joan Boyes

**Affiliations:** School of Molecular and Cellular Biology, Faculty of Biological Sciences, University of Leeds, Leeds LS2 9JT, UK; School of Molecular and Cellular Biology, Faculty of Biological Sciences, University of Leeds, Leeds LS2 9JT, UK; School of Molecular and Cellular Biology, Faculty of Biological Sciences, University of Leeds, Leeds LS2 9JT, UK; School of Molecular and Cellular Biology, Faculty of Biological Sciences, University of Leeds, Leeds LS2 9JT, UK; School of Molecular and Cellular Biology, Faculty of Biological Sciences, University of Leeds, Leeds LS2 9JT, UK

## Abstract

Transcription enhancers are essential activators of V(D)J recombination that orchestrate non-coding transcription through complementary, unrearranged gene segments. How transcription is coordinately increased at spatially distinct promoters, however, remains poorly understood. Using the murine immunoglobulin lambda (Igλ) locus as model, we find that three enhancer-like elements in the 3′ Igλ domain, Eλ3–1, HSCλ1 and HSE-1, show strikingly similar transcription factor binding dynamics and close spatial proximity, suggesting that they form an active enhancer hub. Temporal analyses show coordinate recruitment of complementary V and J gene segments to this hub, with comparable transcription factor binding dynamics to that at enhancers. We find further that E2A, p300, Mediator and Integrator bind to enhancers as early events, whereas YY1 recruitment and eRNA synthesis occur later, corresponding to transcription activation. Remarkably, the interplay between sense and antisense enhancer RNA is central to both active enhancer hub formation and coordinate Igλ transcription: Antisense Eλ3–1 eRNA represses Igλ activation whereas temporal analyses demonstrate that accumulating levels of sense eRNA boost YY1 recruitment to stabilise enhancer hub/promoter interactions and lead to coordinate transcription activation. These studies therefore demonstrate for the first time a critical role for threshold levels of sense versus antisense eRNA in locus activation.

## Graphical Abstract

**Graphical Abstract:**
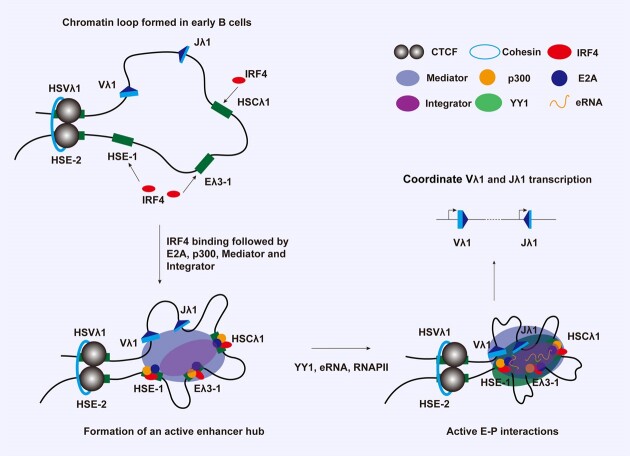

## INTRODUCTION

The spatiotemporal control of gene transcription is a highly intricate and tightly regulated process that is crucial for eukaryotic development. Gene transcription requires regulatory events at promoters, where transcription factors bind to specific motifs that lie upstream of the transcription start site (TSS), to activate assembly of the RNA polymerase II (RNAPII) pre-initiation complex (1). Whilst these promoter-specific events are important to control basal transcription activity, much greater regulation stems from a second, more abundant class of regulatory element, namely transcription enhancers (2). These can reside many thousands of bases from the cognate gene promoters, either upstream or downstream, and are composed of concentrated clusters of recognition motifs for diverse transcription factors, often including nucleosome-binding factors, architecture factors and transcription coactivators (3). Transcription enhancers physically interact with their target gene promoters to vastly increase the level at which the gene is transcribed (4). Functional enhancer–promoter contacts are strongly influenced by the way in which chromosomes are folded in three–dimensional space (5); the latter occurs in a hierarchical manner to give compartments, topologically associating domains (TADs) and insulated neighbourhood domains (INDs), that are thought to represent structural and functional units of genome organization. Physical contacts between different *cis-*acting elements across structural unit boundaries are relatively infrequent whereas efficient tissue-specific gene expression requires transcriptional enhancers and their cognate promoters to be constrained within the same genome structural unit (6).

Antigen receptor loci are essential to generate a highly diverse adaptive immune system. These loci, however, present a unique problem for enhancer-mediated gene activation: Generation of antigen receptor diversity requires recombination between complementary gene segments that can be many kilobases to megabases apart. These gene segments must be coordinately activated via non-coding transcription to increase their accessibility prior to recombination (7,8). Enhancers are central to regulating this non-coding transcription (9) but how enhancers coordinately activate promoters that are far apart in the primary sequence, is poorly understood. This problem is exacerbated by the presence of up to 100 gene segments and many potential regulatory elements in some loci. Since the appropriate chromatin environment is a prerequisite to facilitate enhancer–promoter interactions, initial studies focused on chromatin folding of the IgH and Igκ loci, using DNA fluorescence *in situ* hybridization (FISH) and 3C derivative technologies (10–14). From this, it was proposed that prior to V(D)J recombination, antigen receptor loci form a poised state where they are contracted via a series of loop domains. Contraction is tightly associated with binding of histone modifiers, lymphocyte-specific transcription factors and architecture factors, such as p300, IRF4, PAX5, E2A, CTCF, cohesin and YY1 at interspersed DNA regulatory elements throughout the locus and correlates with enhanced non-coding transcription of unrearranged gene segments (15–20). However, these studies did not explore the regulation of antigen receptor locus activation and chromatin folding in fine detail. Indeed, whilst analysis of chromatin folding in B-cells at different stages of development enables predictions regarding the coordination of events, these studies cannot truly unravel the temporal order of locus specific enhancer–promoter communications and coordinated activation in any detail.

A barrier to the temporal analysis of coordinate locus activation has been the lack of a homogenous population of lymphocytes in which antigen receptor locus activation can be induced. Activation of light chain loci is a hallmark of the pro-B to pre-B transition and previous studies showed that Igλ locus activation absolutely relies on the Eλ3–1 enhancer (21). Notably, this enhancer contains binding sites for the transcription factor, IRF4 and we showed that remarkably, equipping with pro-B cells with pre-B levels of just this single transcription factor, is sufficient to completely activate transcription and recombination of unrearranged Igλ gene segments (7). This, together with the small size of the murine Igλ locus, spanning just ∼230 kb, and low number of functional gene segments provides an excellent system to temporally dissect locus activation. Therefore, to unravel the regulation of enhancer–promoter interactions and changes in chromatin organization required for coordinate V and J gene segment activation, we developed transgenic mice and a pro-B cell line that expresses an inducible IRF4. By studying the dynamics of transcription factor recruitment and changes in Igλ chromatin organization, we built a detailed picture of the stages of activation and show that coordinate transcription factor binding to three enhancer-like elements is essential to form an active enhancer hub. This hub then coordinately activates transcription through V and J gene segments. Remarkably, the interplay between sense and antisense enhancer RNAs (eRNAs) is central Igλ activation: Threshold levels of sense versus antisense eRNA are vital to control YY1 recruitment, stabilisation of enhancer hub formation and enhancer–promoter interactions, and lead to high levels of Igλ non-coding transcription.

## MATERIALS AND METHODS

### Biological resources

The plasmids listed were obtained from Addgene (catalogue numbers in brackets) and were kind gifts from the individuals shown: LentiCRISPR v2 and lenti-sgRNA-MS2-zeo (Feng Zhang, #52961 & #61427); pLKO.1-TRC (David Root, #10878); pCMVR8.74 and pMD2.G (Didier Trono, #22036 & #12259); MSCV-IRES-GFP (Tannishtha Reya, #20672). pGL3-Jλ1p was constructed by cloning the Jλ1 promoter (chr16:19063354–19064105) in front of luciferase reporter gene in pGL3-Basic (Promega). To construct pGL3-Jλ1p-Eλ3–1, the Eλ3–1 enhancer (chr16: 19026931–19027772) was cloned ∼3 kb upstream of the Jλ1 promoter in pGL3-Jλ1p. pRC-IRF4ER was generated by fusing the human oestrogen receptor hormone binding domain from MyoD-ER to the N-terminus of *Irf4* and cloning into pRC/CMV (Invitrogen). MSCV-IRF4-ER-IRES-GFP was constructed by sub-cloning IRF4ER from pRC-IRF4ER into the blunted EcoRI and XhoI sites of MSCV-IRES-GFP. To avoid activation of IRF4-ER by estrogenic compounds within culture medium and to increase the sensitivity to 4-OH tamoxifen, point mutations M543A/L544A (MSCV-IRF4-ERT2-IRES-GFP) were introduced into the ER domain (22) by Q5® site directed mutagenesis. shRNA sequences that target *Med23*, *Med1*, *Yy1*, *Spi1*, sense Eλ3–1 and antisense Eλ3–1 eRNAs were obtained from The RNAi Consortium database (TRC, Broad Institute) and cloned into pLKO.1-TRC. Signal guide (sg)RNAs targeting Eλ3–1 and HSE-1 were designed using the online design software (http://crispr.mit.edu) and cloned into the lentiCRISPR v2 or lenti-sgRNA-MS2-zeo. pLKO-sEλ3–1e and pLKO-asEλ3–1e were generated by replacing the shRNA cassette with sEλ3–1e and asEλ3–1e genomic sequences in pLKO.1-TRC, respectively. pLKO-T7p-sEλ3–1e and pLKO-T7p-asEλ3–1e were constructed by replacing the U6 promoter with the T7 promoter in pLKO-sEλ3–1e and pLKO-asEλ3–1e.

Non-transgenic mice (CBA/C57BL/6J) were obtained from the University of Leeds animal facility. IRF4-ER transgenic mice were generated in the same way as the PIP2, PIP3 and PIP4 transgenic mice described previously (7) where *Irf4* was expressed under control of the pro-B cell specific λ5 promoter and LCR. Here, IRF4 was fused to the estrogen receptor hormone binding domain and the fusion gene substituted for *Irf4* in the λ5 promoter/ LCR cassette. Animals were sacrificed at 5–7 weeks, bone marrow was removed from femurs and used for the isolation of pro- or pre-B cells by flow cytometry. Equivalent numbers of male and female animals were used overall. All animal procedures were performed under Home Office licence PPL 70/7697 and P3ED6C7F8, following reviews by the University of Leeds ethics committee. They were housed in a full barrier facility, with no more than six animals per cage, where all mice are free of common pathogens, including murine norovirus, Pasteurella and Helicobacter.

HEK293T were a kind gift from Prof. Mark Harris and Phoenix cells were generously supplied by Dr Garry Nolan. They were maintained in Dulbecco's Modified Eagle Medium (DMEM) supplemented with 10% foetal calf serum, 4 mM l-glutamine, 50 U/ml penicillin and 50 μg/ml streptomycin. Cells were grown in a humified incubator at 37°C with 5% CO_2_.

103/BCL-2 (a kind gift from Prof. Naomi Rosenberg) and PIPER-15 cells were maintained, at a density of 0.5–2 × 10^6^ cells/ml, in complete Roswell Park Memorial Institute (RPMI)-1640 medium supplemented with 10% foetal calf serum, 4 mM L-glutamine, 50 U/ml penicillin and 50 μg/ml streptomycin and 50 μM β-mercaptoethanol. Cells were grown at 33°C with 5% CO_2_.

Pro-B cells were flushed from the femurs of 5–7 week old mice and cultured in pro-B cell medium as described previously (7). Primary cells were cultured at 33°C, 5% CO_2_ for 7 days with the addition of 5 ml fresh medium after 4 days.

### Generation of A-MuLV-transformed pro-B cell lines

The AB010 cell line (23), which secretes Abelson murine leukaemia virus (A-MuLV), was grown for two days past confluency in supplemented DMEM. The virus containing supernatant was removed and concentrated using a Centricon Plus-70 centrifugal device. Bone marrow was flushed from the femurs of 5–7-week-old mice and cells were immediately infected with A-MuLV. Red blood cells were lysed for ten min by suspension in 168 mM NH_4_Cl. Infection with A-MuLV was performed by the addition of 1 ml of primary cells at a concentration of 2 × 10^6^ cells/ml to 1 ml of concentrated viral supernatant, in the presence of 8 μg Polybrene (Millipore). Cells were incubated at 37°C for 2.5 h with agitation every 20 min and plated at concentrations of 1 × 10^6^ cells/ml in semi-solid agar (RPMI supplemented with 20% foetal calf serum, 2 mM l-glutamine, 50 μg/ml streptomycin, 50 U/ml penicillin, 50 μM β-mercaptoethanol and 0.3% bacterial agar (Oxoid Ltd). Following infection, cells were maintained in a humidified atmosphere at 37°C, adding 1 ml of semi-solid agar every 4 days.

### Generation of MSCV-IRF4-ERT2 cell lines

Retroviruses, produced using the MSCV-IRF4-ERT2-IRES-GFP construct, were transduced into the A-MuLV immortalized pro-B cell line. Infection was monitored via GFP expression and flow cytometry. To generate monoclonal cell lines, 1 × 10^4^ cells expressing the highest level of GFP were purified by flow cytometry and plated in 10 ml of semi-solid agar. After 10 days, macroscopic colonies were transferred to RPMI in 24-well plates and expanded.

### Tamoxifen and Imatinib treatment of cell lines

The IRF4-ERT2 protein was activated in PIPER-15 cells by addition of tamoxifen. Inductions were performed by resuspending 1–5 × 10^6^ cells at 0.5 × 10^5^ cells/ml in RPMI; 4-hydroxytamoxifen (Insight Biotechnology; HY-16950) was added to a final concentration of 2 μM. Cells were incubated at 37°C with 5% CO_2_ for the times indicated. Imatinib was added to PIPER-15 cells, resuspended as above, at final concentrations of 1–100 nM. Cells were incubated for 48 h prior to harvest.

### Preparation of whole cell and nuclear extracts

Whole cell extracts were prepared by washing cells with PBS and resuspending at 2 × 10^4^ cells/ml in a 3:1 mix of RIPA (25 mM Tris pH 8.2, 50 mM NaCl, 0.5% NP-40, 0.5% sodium deoxycholate, 0.1% SDS) and lysis buffer (5% SDS, 0.15 M Tris pH 6.7, 30% glycerol), supplemented with protease inhibitors (Complete™, Mini Inhibitor Cocktail Tablets, Roche). Samples were boiled for 5 min and centrifuged at 16 000 g for 10 min at 4°C.

Nuclear extracts were prepared by resuspending PBS washed cells at a density of 1 × 10^6^ cells/ml in 1 ml of lysis buffer (10 mM Tris pH 8, 10 mM NaCl, 0.2% NP-40, 50 μg/ml PMSF, 1× Complete™ protease inhibitor cocktail, Roche) and incubating on ice for 20 min. Nuclei were pelleted at 800 g for 2 min before resuspending in 100 μl 1× Laemmli loading buffer and boiling for 5 min.

Whole cell and nuclear extracts were either used immediately for western blotting or flash frozen on dry ice and stored at –80°C until required.

### Western blotting

Following electrophoresis, proteins were transferred to PVDF membrane (Immobilon-P, IPVH00010, Millipore) in a Trans-Blot Turbo transfer system (Bio-Rad) for 30 min at 25 V. The PVDF membrane was blocked in a solution of 5% non-fat milk powder in TBS-T (50 mM Tris pH 7.6, 150 mM NaCl, 5% milk, 0.05% Tween-20) for 1 h at room temperature. All primary antibody hybridisations were conducted overnight at 4°C, whereas secondary or tertiary antibody hybridisations were performed at room temperature for an hour. Antibodies are given in Supplementary Table 1 and were used at the dilutions recommended by the manufacturer. After each hybridisation, membranes were washed with TBS-T, with changes every 5 min for 1 h. Membranes were developed by incubation with enhanced chemiluminescence substrate (Thermo Scientific) for 2 min at room temperature and imaged using a G:BOX ChemiXT4 system (Syngene).

### Total RNA extraction and reverse transcription

Total RNA was extracted from approximately 2 × 10^6^ cells using TRIzol (Invitrogen #3289) according to the manufacturer's instructions, followed by treatment with 2 U DNase I (Worthington) for 1 hr at 37°C in 100 μl of 1 x NEB DNase I buffer (10 mM Tris pH 7.5, 2.5 mM MgCl_2_, 0.5 mM CaCl_2_). Following phenol-chloroform extraction and ethanol precipitation, RNA concentration was determined using a DS11 + spectrometer (DeNovix).

1 μg of RNA was reverse transcribed with M-MuLV reverse transcriptase (Invitrogen). Briefly, 1 μg of RNA was added to 2.5 μM oligo dT primer (or strand-specific primer, where noted), 500 μM dNTPs and ddH_2_O to give a total volume of 12 μl. This was incubated at 65°C for 5 min and immediately placed on ice before addition of 4 μl first strand buffer (Invitrogen), 10 mM DTT and 1 μl RNasinPlus (Promega). The reaction was incubated at 37°C for 2 min, followed by addition of 1 μl Moloney-Murine Leukaemia Virus Reverse Transcriptase (Invitrogen), incubation at 37°C for 50 min prior to heat inactivation at 70°C for 15 min.

### Real-time PCR using SYBR Green

Quantitative PCR was performed using a Corbett Rotor-Gene 6000 machine and analysed using the Corbett Rotor-Gene 6000 Series Software (v.1.7, build 87). A typical qPCR reaction contained 5 μl 2 × SensiFAST SYBR No-Rox mix (Bioline #BIO-98080), 2–10 ng DNA template, or cDNA at a final dilution of 1:100, 400 nM of each primer in a total volume of 10 μl. Primer sequences are given in Supplementary Table 1. All reactions were performed in duplicate. In each case, a standard curve of the amplicon was analysed concurrently to evaluate the amplification efficiency and to calculate the relative amount of amplicon in unknown samples. R^2^ values were 1 ± 0.1. A typical cycle consisted of: 95°C for 3 min, followed by 40 cycles of 95°C for 5 s, Tm for 10 s and 72°C for 10 s, where *T*_m_ = melting temperature of the primers. A melt curve, to determine amplicon purity, was produced by analysis of fluorescence as the temperature was increased from 72°C to 95°C. Amplicons were 100–200 bp.

### Analysis of Vλ1-Jλ1 recombination

Primary pro-B cell cultures from IRF4-ER transgenic mice were expanded for seven days, as described (7). Tamoxifen was added to at a final concentration of 2 μM for the induction times indicated, prior to cell harvest. Pro-B cells were then purified by flow cytometry with 2 μM Tamoxifen present in all buffers and DNA was prepared as described (7), using at least four phenol/chloroform extractions to remove contaminants prior to ethanol precipitation. The resuspended DNA was quantified using a Quant-iT™ PicoGreen™ assay (Invitrogen), according to the manufacturer's instructions. DNA amounts were further normalised using 2–3 ng in qPCR reactions and Intgene III primers. Vλ1/Jλ1 recombination was determined via nested qPCR, using 3 ng DNA and 15 cycles in the first round of PCR. Following a 10-fold dilution of the product, 1.5 μl was used in the second round qPCR reaction. Primer sequences are given in Supplementary Table 1.

### Transfection of HEK293T and Phoenix cells

Transfection of HEK293T and Phoenix cells was carried out using PEI (Alfa Aesar #043896.01). Twenty-four hours before transfection, 3 × 10^6^ cells were plated per 10 cm dish in complete DMEM. Three hours prior to transfection, the medium was changed to fresh complete DMEM medium. Plasmid DNA (10 μg) was mixed well with 500 μl OptiMEM™ by gentle vortexing. Concomitantly, 30 μl of PEI solution (1 mg/ml) was diluted with 500 μl of OptiMEM medium. The solutions were then mixed well for 15 s, followed by incubation at room temperature for 15 min. The mixture was added to cells dropwise; cells were then incubated at 37°C for 48 h prior to harvest.

### Transfection of 103/BCL-2 cells

Electroporation was carried out using the Nucleofector™ Kit (LONZA # VPA-1010) according to manufacturer's instructions. Briefly, 4 × 10^6^ cells were washed twice with ice cold PBS and resuspended in 100 μl of transfection reagent (82 μl nucleofector plus 12 μl supplement 2), followed by addition of 2 μg plasmid DNA. Cells were then transferred to a cuvette and electroporated at setting Z01 of the AMAXA electroporator. Following addition of 500 μl complete RPMI medium, cells were decanted into a 6-well plate using a sterile pastette; an additional 1400 μl of RPMI medium was added, followed by incubation at 33°C overnight. Twenty hours prior to harvest, cells were temperature shifted to 39.5°C to inactivate the temperature-sensitive v-Abl kinase (24) and trigger light chain transcription.

### Luciferase reporter assay

The luciferase assay was carried out using the Dual-Luciferase Kit (Promega) according to manufacturer's instructions. Cells were washed twice with ice cold PBS and resuspended in 1 ml Passive Lysis Buffer, followed by gentle shaking at room temperature for 15 min. Following transfer to a fresh Eppendorf tube, the lysate was vortexed vigorously for 15 s and centrifuged at 16 000 g for 10 min at 4°C. 100 μl of the Luciferase Assay substrate was pre-dispensed into a luminometer tube. 20 μl of the lysate was added, followed by determination of firefly luciferase activity using the SIRIUS luminometer v3.0. Renilla luciferase activity was measured by addition of 100 μl of Stop & Glo™ reagent.

### Flow cytometry

Primary pro-B and pre-B cells were stained with FITC and PE conjugated antibodies as described (7). Antibody labelled cells or GFP expressing cells were purified by flow cytometry using a FACSMelody™ cell sorter (Becton Dickinson). GFP expressing cells were analysed by flow cytometry using a CytoFLEX flow cytometer (Beckman Coulter, USA) to determine the percentage of cells that had successfully been transduced. Cells were prepared for flow cytometry by washing with, and resuspension in, ice cold PBS.

### Production of retroviral particles

Retroviral particles were generated using Phoenix cells (25). Twenty-four hours before transfection, 3 × 10^6^ Phoenix cells were plated per 10 cm dish in complete DMEM. Three hours prior to transfection, the medium was changed to DMEM supplemented with 5% foetal calf serum, 4 mM l-glutamine. 4 μg of MSCV-IRF4-ERT2-GFP construct was mixed with 500 μl of OptiMEM by gentle vortexing. Concomitantly, 12 μl of PEI (1 mg/ml) was diluted with 500 μl of OptiMEM. The solutions were then mixed with gentle vortexing for 15 s, followed by incubation at room temperature for 15 min and dropwise addition to cells. Cells were incubated at 37°C for 48 and 72 h prior to harvest. The retrovirus-containing supernatant was filtered through a 0.45 μm syringe filter, flash frozen on dry ice and stored at –80°C until use.

### Production of lentiviral particles

Lentiviral particles were produced in HEK293T cells by transfection with the lentiviral backbone constructs, packaging construct (pCMVR8.74) and envelope construct (pMD2.G). For lentiviral backbone constructs, pLKO.1-TRC was used to produce shRNA-mediated knock-down lentiviral particles. 3 × 10^6^ HEK293T cells were plated per 10 cm dish in complete DMEM 24 h before transfection. Three hours prior to transfection, the medium was changed to DMEM supplemented with 5% foetal calf serum, 4 mM l-glutamine. Separately, 4.9 μg of pLKO.1 shRNA plasmid, 2.6 μg of pCMVR8.74 and 2.5 μg of pMD2.G were mixed with 500 μl of OptiMEM medium by gentle vortexing, whereas 30 μl of PEI stock solution (1 mg/ml) was diluted with 500 μl of OptiMEM medium. Transfection, harvest and storage of lentiviruses was then as described for retroviruses above.

### Knockdown of *Med23*, *Med1*, *Yy1, Spi1* and eRNAs

pLKO.1, expressing the appropriate shRNA, was co-transfected into HEK293T cells with the packaging plasmids, pCMVR8.74 and pMD2.G, to produce lentiviral particles. The resulting lentivirus was used to transduce PIPER-15 cells by spin-fection via centrifugation at 800 g for 30 min at 32°C. After 48 h, puromycin (Cayman Chemical) was added at a final concentration of 2 μg/ml, followed by incubation at 37°C for 7 days.

### Knockout of Eλ3-1 and HSE-1 enhancers

Two CRISPR sgRNA-specifying oligonucleotides that flank the PU.1/IRF4 sites in each enhancer element (Eλ3–1 and HSE-1) were designed as above. Eλ3–1 gRNA1 / HSE-1 gRNA1 oligonucleotides were annealed and cloned into lenti-CRISPR v2 whereas Eλ3–1 gRNA2 and HSE-1 gRNA2 oligonucleotides were cloned into lenti-sgRNA-MS2-zeo. Lentiviral production was performed as described above. Transductions of PIPER-15 cells were performed in a sequential manner. 5 × 10^5^ PIPER-15 cells were spin-fected with 500 μl of Eλ3–1 gRNA2 or HSE-1 gRNA2 lentiviruses; transduced cells were selected with 100 μg/ml Zeocin (Alfa Aesar J67140) after 48 h. After one week of selection, cells were spin-fected with Eλ3–1 gRNA1 or HSE-1 gRNA1 lentivirus and selected for one week with 0.25 μg/ml puromycin. Monoclonal cell lines were generated using semi-solid agar and clones were screened for knockouts by PCR using the primers HSE-1delF/R and Eλ3–1del F/R (Supplementary Table 1). Monoclonal cell lines with apparent deletions in these regions were amplified using the above primers; the products were cloned and knockout of the respective region confirmed by Sanger sequencing.

### Chromatin Immunoprecipitation (ChIP)

ChIP in primary pro- and pre-B cells was carried out according to Boyd and Farnham (26) with modifications using 2 × 10^7^ cells per experiment. ChIP experiments in PIPER-15 cells and with anti-E2A and anti-MED1 antibodies in primary pro- and pre-B cells, were performed according to Nowak *et al.* (27) by first cross-linking with 2 mM Disuccinimidyl Glutarate (DSG, Sigma 80424) and then with 1% formaldehyde. The antibodies and dilutions used are given in Supplementary Table 1. The recovered DNA was analysed using quantitative PCR and the primers shown in Supplementary Table 1.

### Chromatin conformation capture (3C)

3C was carried out according to Dekker *et al* (28) with modifications. 1 × 10^7^ PIPER-15 cells were used per experiment and following preparation of cross-linked nuclei, samples were flash frozen in liquid nitrogen and stored at –80°C. Stored nuclei were resuspended in 500 μl 1.2× NEB Dpn II buffer (50 mM Bis–Tris–HCl pH 6.0, 100 mM NaCl, 10 mM MgCl_2_, 1 mM DTT) in a screw capped tube. SDS was added to a final concentration of 0.3% followed by vigorous pipetting. The nuclei were shaken at 200 rpm for 60 min at 37°C with pipetting every 15 min, to prevent aggregation, prior to addition of Triton X-100 to a final concentration of 3%, and incubation at 37°C for 60 min with shaking. The nuclei were then digested by addition of 100 units of Dpn II (NEB, R0543M) and incubation at 37°C for 4 h with shaking, followed by an overnight digestion with an additional 100 units of Dpn II. Following addition of a further 100 units of Dpn II and incubation for 4 h at 37°C, the restriction enzyme was inactivated by incubation at 65°C for 20 min, and digested nuclei transferred to a fresh tube. Ligation was performed in 7 ml of 1× ligase buffer (50 mM Tris–HCl pH 7.5, 10 mM MgCl_2_, 1 mM ATP, 5 mM DTT) with 25 U T4 DNA ligase (Roche) at 16°C overnight. RNase A was then added to a final concentration of 10 μg/ml at 37°C for 30 min; crosslinks were reversed by addition of proteinase K to a final concentration of 100 μg/ml and incubation at 65°C for at least 4 h. Ligated DNA sample was phenol/chloroform extracted, precipitated with ethanol, and resuspended in 100 μl of TE.

### Preparation of BAC template for 3C analysis

Bacterial artificial chromosome (BAC) Rp23-24i11 was obtained from Children's Hospital Oakland Research Institute and contains the 3′ half of the murine Igλ locus. Dpn II (NEB, R0543M) is blocked by Dam methylation; therefore, BAC DNA was digested with its isoschizomer Sau3AI (NEB, R0169S) and ligated at high concentration to generate all possible ligation products as a 3C normalisation control. 20 μg of BAC DNA was treated with 25 U of Sau3AI in a total volume of 500 μl at 37°C overnight. The digested BAC DNA was phenol–chloroform extracted, recovered by ethanol precipitation and resuspended in 40 μl TE. BAC DNA was ligated with 2000 cohesive end units/ml of T4 DNA ligase in a total volume of 60 μl at 16°C overnight. The ligated products were phenol/chloroform extracted, ethanol precipitated and resuspended in 100 μl of TE.

### Nested PCR assay to detect 3C interactions

Eλ3–1 was used as a viewpoint to determine interactions within the Igλ locus. A nested PCR assay was used to detect 3C interactions between Eλ3–1, HSE-1 and other *cis-*acting elements using the primers given in Supplementary Table 1. Nested PCR reactions were also performed on the BAC control template to correct for differences in primer efficiency. The first round of PCR was performed using Taq DNA polymerase. For the second round, TaqMan qPCR was conducted in duplicate in 10 μl final volume with 5 μl of 1:10 diluted first round PCR product, 400 pM each primer, 100 pM 5′ nuclease probe and 5 μl qPCRBIO probe mix (PCRBIO PB20.21–05). For Supplementary Figures 1A, B and 4G, H, only a single round of qPCR was performed, using TaqMan probes and the primers given in Supplementary Table 1; HSE.1 was used as an additional viewpoint in Supplementary Figures 1B and 4G. All 3C samples were normalised by analysis of interactions in the *Ercc3* locus which is expected to be consistent across all cell types (29).

### *In vitro* transcription of enhancer RNAs

pLKO-T7p-sEλ3–1e, pLKO-T7p-asEλ3–1e and pLKO-T7p-randomRNA were linearized with EcoRI which cleaves just downstream of the respective eRNA sequences. These were used as templates for *in vitro* transcription of sEλ3–1e, asEλ3–1e and random RNAs with T7 RNA polymerase (NEB, M0251S), according to the manufacturer's instructions. The *in vitro* transcribed products were treated with DNaseI to digest the template DNAs, ethanol precipitated and resuspended in DEPC-treated deionized water.

### Electrophoresis of enhancer RNAs

Agarose gel electrophoresis of enhancer RNAs was conducted as described previously (30). Briefly, 1 μg of enhancer RNA was heated at 95°C for 2 min and placed on ice for 2 min. RNAs were incubated at 37°C for 2 h and then mixed with native loading buffer (10× stock: 15% ficoll, 0.25% bromophenol blue, and 0.25% xylene cyanol FF) before loading onto a 1% agarose gel in TAE. Electrophoresis was at 40 V for 1.5 h at 4°C.

### RNA immunoprecipitation (RIP)

RIP was performed according to (31). The IgG and YY1 antibodies used are given in Supplementary Table 1. The recovered RNA was reversed transcribed with strand specific primers and then analysed using quantitative PCR and the primers shown in Supplementary Table 1.

### ATAC-seq

ATAC-seq was performed as described previously (32) with minor modifications. Briefly, 5 × 10^4^ cells were pelleted at 300 g for 5 min, washed with 50 μl PBS and pelleted for 5 min at 300 g. Cells were lysed by resuspension in 50 μl of ATAC-seq RSB (10 mM Tris–HCl pH 7.4, 10 mM NaCl and 3 mM MgCl_2_) containing 0.1% NP40, 0.1% Tween-20 and 0.01% digitonin and incubation on ice for 3 min. Nuclei were washed to remove contaminating mitochondria with 1 ml of RSB containing 0.1% Tween-20 and pelleted at 500 g for 10 min. Nuclei were then resuspended in 50 μl of transposition mix (25 μl 2× TD buffer, 2.5 μl transposase, 16.5 μl PBS, 0.5 μl 1% digitonin, 0.5 μl 10% Tween-20 and 5 μl water) and incubated on a thermomixer at 37°C for 30 min at 900 rpm. Reactions were purified using a Qiagen MinElute PCR-purification column. Library preparation was performed as described previously (33) with 10 cycles of amplification and purification using a Qiagen MinElute PCR-purification column. Samples were paired-end sequenced by Novogene on a NovaSeq 6000 S4 flow cell with a read length of 150 bp.

### Analysis of next generation sequencing data

Accession numbers of all datasets used, are given in Supplementary Table 1.

### ChIPseq

Read files in FASTQ format were downloaded from the European Nucleotide Archive (ENA; https://www.ebi.ac.uk/ena) and sequencing adapters were removed by TrimGalore (0.5.0). Reads were aligned to the Mus musculus (mm9) genome using Bowtie2 (2.3.4.2) and default parameters; multimapping reads as well as poor quality alignments were removed using Samtools (1.9). Peaks were called using MACS2 (2.1.0), for transcription factors, using default parameters. Visualisation was performed using the Integrated Genome Browser IGV (2.4.2) after converting the bedgraph output from MACS2 into a binary ‘tiled’ format using IGV tools (2.3.98).

### ATAC-seq

Read files were downloaded from the ENA, trimmed and aligned as above, the Bowtie2 (2.3.4.2) max insert parameter (–X) was set to 2000 to enable the mapping of large inserts that are typical of ATAC-seq. Multimapping reads were removed by Samtools (1.9) before peak calling. ATAC-seq peaks were called by MACS2 (2.1.0) with the parameters –nomodel –shift 150 –extsize 300.

For new ATAC-seq data: Following quality checking of FASTQ files by FastQC v0.12.1, reads were trimmed and aligned as above. PCR duplicates were removed using Picard MarkDuplicates v3.0.0. Problematic genomic regions present in the ENCODE Blacklist (34) were removed from the aligned files and further quality control of the aligned files was performed using Samtools v1.17. The deep learning based peak caller LanceOtron v1.0.8 (with a peak score cut-off value of 0.5) was used to call peaks. BigWigs were generated using the deepTools (v3.5.1) bamCoverage command, with the flags –extendReads –normalizeUsing RPKM, and visualized in the UCSC genome browser.

### Hi-C

Read files (FASTQ) were downloaded and trimmed as above, before being aligned separately to the mm9 genome using Bowtie2 (2.3.4.2). The HOMER program (4.9) makeTagDirectory was used to process the aligned reads into a tag directory for downstream analysis. Significant interactions occurring in the Igλ locus were identified with the HOMER script analyzeHiC. This command was run with the following parameters: -res 10000 -interactions < interaction_file> -pos < region of interest> –center. This script identifies and reports pairs of regions that have a significantly increased number of interactions than would be expected from the background model. The ‘center’ argument re-centres the regions outputted to the average of the position of the Hi-C reads participating in the interaction. Visualisation of the Hi-C interactions was performed using Circos (0.69).

### Statistical analyses

Statistical analyses were performed using GraphPad Prism v9. Analyses of fold changes between biological replicates, using biologically distinct samples from the same types of cells, were performed using a paired Student's *t* test where *P < 0.05, **P < 0.01, ***P < 0.001, ****P < 0.0001.

## RESULTS

### PU.1 and IRF4 binding to the Eλ3-1 enhancer activates Igλ gene transcription

**Figure 1.**
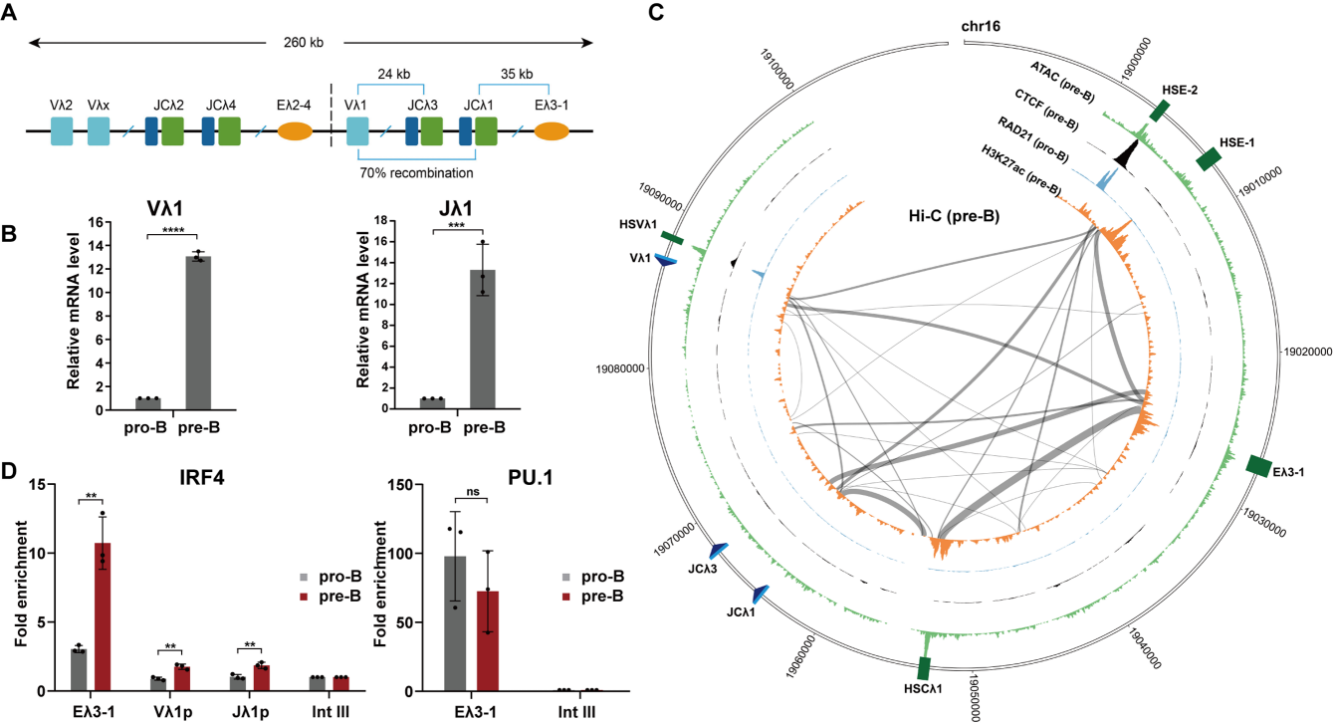
The enhancer, Eλ3–1, activates target gene transcription via PU.1 and IRF4. (**A**) Schematic of the murine Igλ locus; the potential gene duplication is indicated by the dashed line. Constant exons (C) are depicted by green rectangles; V gene segments by cyan rectangles; J gene segments by blue rectangles and enhancers by orange ovals. 70% of recombination occurs between the Vλ1 and Jλ1 gene segments. (**B**) Transcription levels of Vλ1 and Jλ1 in primary mouse pro-B and pre-B cells, determined by qPCR. Data are normalized to *Hprt* expression levels. (**C**) Schematic of significant Hi-C interactions in the 3′ half of the murine Igλ locus. CTCF, RAD21, H3K27ac ChIP-seq, Hi-C and ATAC-seq from pre-B cells were analysed using the HOMER software package and visualised using Circos (RAD21 data from pro-B cells). Significant interactions in 10 kb windows are shown. (**D**) IRF4 and PU.1 binding was analysed by ChIP-qPCR in primary mouse pro-B and pre-B cells. The fold enrichment over input at Eλ3–1, Vλ1, Jλ1 and Intgene III (negative control region) is shown. All values are normalized to binding at the Intgene III negative control. Error bars show standard error of the mean (SEM) from three biological replicates.

The relatively simple organisation of the small murine lambda light chain locus offers an excellent system to dissect temporal and coordinate activation of antigen receptor loci. This locus is thought to have arisen by an evolutionary duplication event (35), resulting in similarly organised 5′ and 3′ domains, each with only 3–4 gene segments and several DNA regulatory elements (Figure 1A). Just like other antigen receptor loci, the V and J gene segments are many kb apart but crucially, ∼70% of Igλ recombination occurs between Vλ1 and Jλ1 (36). Therefore, the mechanism of coordinate gene segment activation can be investigated by focusing on just these two gene segments. Recombination requires non-coding transcription through the unrearranged gene segments and the B cell specific enhancer, Eλ3–1, is pivotal to this regulation (21,37). Consistent with this, the significant increase in Vλ1 and Jλ1 transcription from pro- to pre-B cells (Figure 1B), correlates with extensive interactions between Eλ3–1 and both Jλ1 and Vλ1, as predicted by Hi-C (Figure 1C) and confirmed by 3C (Supplementary Figures S1A and S1B).

Two lymphocyte-specific transcription factors, PU.1 (38) and IRF4 (39) bind to a composite IRF4/PU.1 site in Eλ3–1 (39,40) and have been shown to be important to its function (41). To further verify this, luciferase constructs were generated with the Jλ1 promoter ± Eλ3–1 sequences and electroporated into the transformed pre-B cell line, 103/BCL-2 (24). Inclusion of Eλ3–1 results in ∼3-fold more luciferase activity compared to the Jλ1 promoter alone whereas single mutations within the core consensus motifs of PU.1 or IRF4 cause a significant decrease or even loss of luciferase activity compared to the wild-type enhancer (Supplementary Figure S1C).

To determine if enhanced Vλ1 and Jλ1 transcription correlates with increased IRF4 binding at Eλ3–1, chromatin immunoprecipitation (ChIP) was performed. Consistent with previous findings (7), IRF4 binding at Eλ3–1 increases ∼3-fold from primary pro- to pre-B cells (Figure 1D). A small, but reproducible, increase in IRF4 binding is also detected at both Vλ1 and Jλ1 promoters in pre-B cells (Figure 1D); IRF4 does not directly bind to these promoters but instead, the observed increase may result from enhancer–promoter interactions. By contrast, ChIP analyses show PU.1 binding at Eλ3–1 does not change significantly between pro- and pre-B cells (Figure 1D). PU.1 has a high affinity for its binding motif, whereas IRF4 interacts only weakly with DNA in the absence of PU.1 (42). From this, and previous studies (24,39,40), it is feasible that PU.1 provides a binding platform for IRF4.

### Induction of the mouse IGλ locus enables temporal investigation of coordinate enhancer-mediated activation

Previous data from our lab showed that equipping pro-B cells with elevated, pre-B cell levels of IRF4 is sufficient to completely trigger Igλ locus activation (7). The ability of just a single transcription factor to cause such profound changes at a small, well-defined locus provides a rare opportunity to follow enhancer-mediated locus activation temporally and gain novel insights into key regulatory events. We therefore generated transgenic mice that express an inducible IRF4, namely IRF4-ER, where the oestrogen receptor hormone binding domain is expressed in frame with IRF4 (Supplementary Figure S1D). Using pro-B cell cultures from these mice, we find that Vλ1 and Jλ1 transcription are coordinately and sharply increased between 7 and 8 h of addition of tamoxifen (Supplementary Figure S1D) whereas recombination begins to increase shortly thereafter and continues to increase until 15 hpi (Supplementary Figure S1E), consistent with the requirement for non-coding transcription to activate recombination (43).

**Figure 2.**
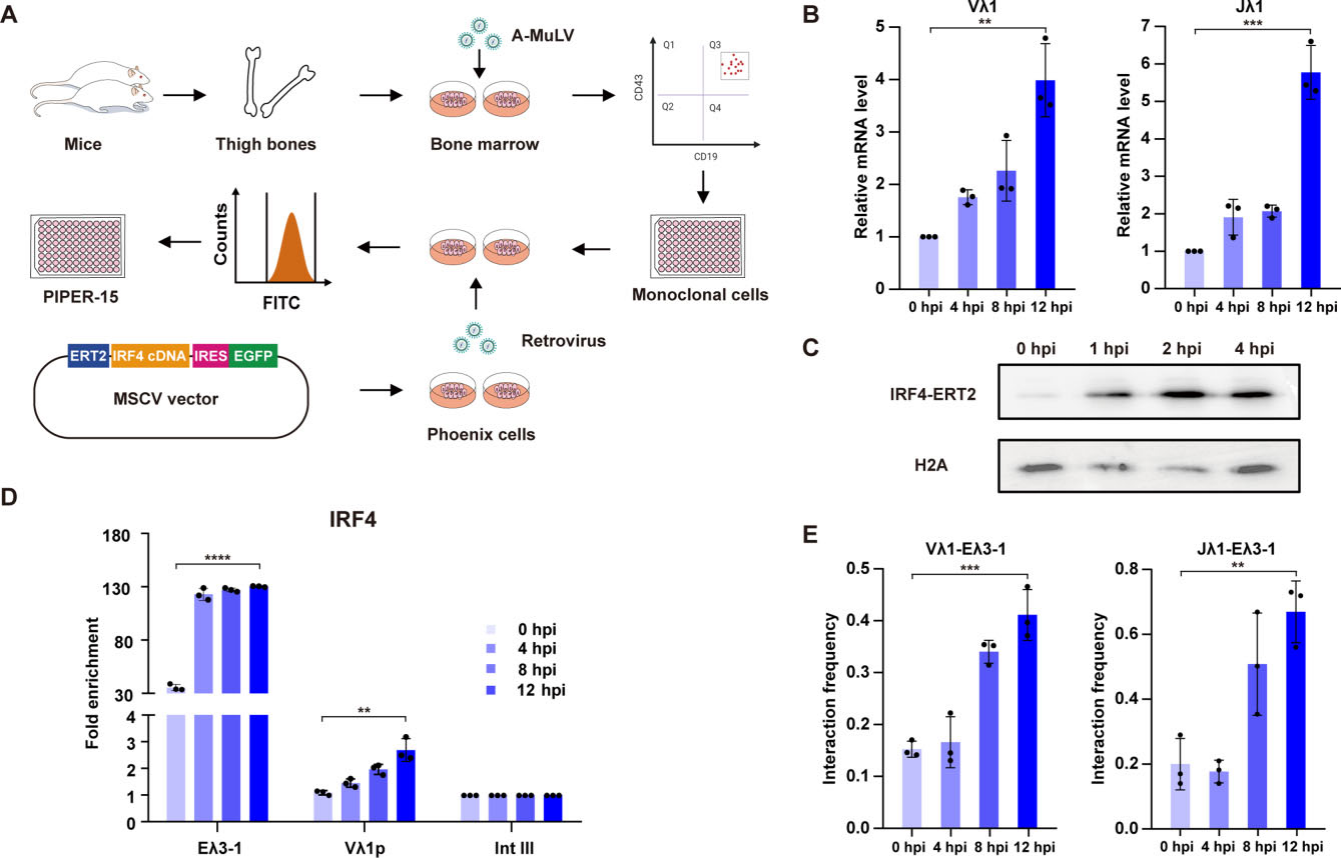
Development of an inducible system to investigate enhancer–promoter interactions. (**A**) Schematic of the generation of the pro-B cell line, PIPER-15, that expresses the inducible transgene, *Irf4-ERT2*. Bone marrow was extracted from six-week-old mice and immediately infected with the Abelson murine leukaemia virus (A-MuLV) for immortalization. Single cells were isolated by flow cytometry using pro-B specific markers, CD19 and CD43. Retroviruses were generated by transfecting the construct, MSCV-IRF4-ERT2-IRES-GFP, into Phoenix cells, followed by transduction of immortalized pro-B cells by spin-fection. Single, transduced pro-B cells with the highest expression of GFP were isolated by flow cytometry. (**B**) The level of Vλ1 and Jλ1 non-coding transcription was analysed by RT-qPCR in PIPER-15 cells following induction of IRF4-ER with 4-hydroxytamoxifen. A sharp increase is observed from 8 to 12 hpi. Data are normalized to *Hprt* expression levels. (**C**) Analysis of IRF4-ERT2 by western blotting in nuclear extracts of PIPER-15 cells following induction with 4-hydroxytamoxifen. Histone H2A levels are used as a loading control. (**D**) IRF4 binding to the Eλ3–1 enhancer and Vλ1 promoter in PIPER-15 cells following induction. The fold enrichment over input at Eλ3–1, Vλ1 and Intgene III (negative control region) is shown. All values are normalized to binding at the Intgene III negative control. (**E**) The interaction between Eλ3–1 and Vλ1 as well as Jλ1 was analysed by 3C-qPCR in PIPER-15 cells following induction. Data were normalized using an interaction within the *Ercc3* locus. Error bars show standard error of the mean (SEM) from three biological replicates.

To have sufficient cells to investigate this activation in more detail, we next generated a pro-B cell line that also expresses inducible IRF4 (IRF4-ERT2; Figure 2A), but where a modified oestrogen receptor hormone binding domain was used to reduce non-specific activation (22). Single cell clones were selected that express IRF4-ERT2 at pre-B cell levels (Supplementary Figure S1F), resulting in the cell line, PIPER-15 (Figure 2A). Temporal RT-qPCR analyses show that addition of the oestrogen antagonist, 4-hydroxytamoxifen, results in a modest increase in Vλ1 and Jλ1 transcription in PIPER-15 cells from 0 to 8 h post-induction (hpi), followed by a sharp increase from 8 to 12 h (Figure 2B); this correlates well with the changes in primary cells, albeit with slightly delayed kinetics. Consistent with its regulatory role, IRF4-ERT2 translocates to the nucleus, reaching its highest level at just 2 h post-induction (Figure 2C). Furthermore, IRF4-mediated activation is dependent on PU.1: knock-down studies show that loss of PU.1 significantly reduces Vλ1 and Jλ1 transcription and IRF4 binding (Supplementary Figures S2A–C).

To investigate the link between activator binding to the enhancer and target gene activation, temporal ChIP analysis was performed. Remarkably, IRF4 binding to Eλ3–1 increases dramatically from 0 to 4 hpi, followed by only a slight increase from 4 to 12 hpi (Figure 2D), suggesting that enhancer binding by IRF4 is an early event in Igλ activation. A limited but clearly detectable increase of IRF4 binding to the Vλ1 promoter is also observed at 8 hpi (Figure 2D) but significant enrichment of IRF4 at the Jλ1 promoter was not detected (Supplementary Figure S2D). Consistent with this, Jλ1 transcription is substantially repressed in PIPER-15 cells compared to primary pre-B cells (Supplementary Figure S2E), even though Vλ1 and Jλ1 show a similar fold-induction (Figure 2B). Reduced Jλ1 transcription may be explained, however, by binding of the transcriptional repressor, STAT5, to the Jλ1 promoter (Supplementary Figure S2F), where STAT5 is likely activated by v-Abl kinase (44) in the Abl-kinase-derived cell line, PIPER-15. Consistent with this, Jλ1 transcription is significantly increased upon addition of imatinib to inhibit Abl-kinase (Supplementary Figure S2G).

Chromatin contraction between an enhancer and its cognate promoter is required for transcription activation and therefore, it would be expected that the interaction frequency between the Eλ3–1 enhancer and Vλ1 and Jλ1 promoters will increase post-induction. Temporal chromatin conformation capture (3C) analysis confirmed that this is indeed the case by 8 hpi, just before enhanced Vλ1 transcription is observed (Figure 2E). This is also consistent with the increased enhancer–promoter contacts observed in primary cells (Supplementary Figures S1A and S1B). Together, these data show a coordinate increase in Vλ1 and Jλ1 transcription as well as striking temporal changes in their interactions with Eλ3–1, implying that PIPER-15 cells are a good model to investigate the mechanisms that underpin coordinate, enhancer-mediated activation.

### IRF4 regulates sequential recruitment of diverse transcription factors to trigger enhancer–promoter interactions

**Figure 3.**
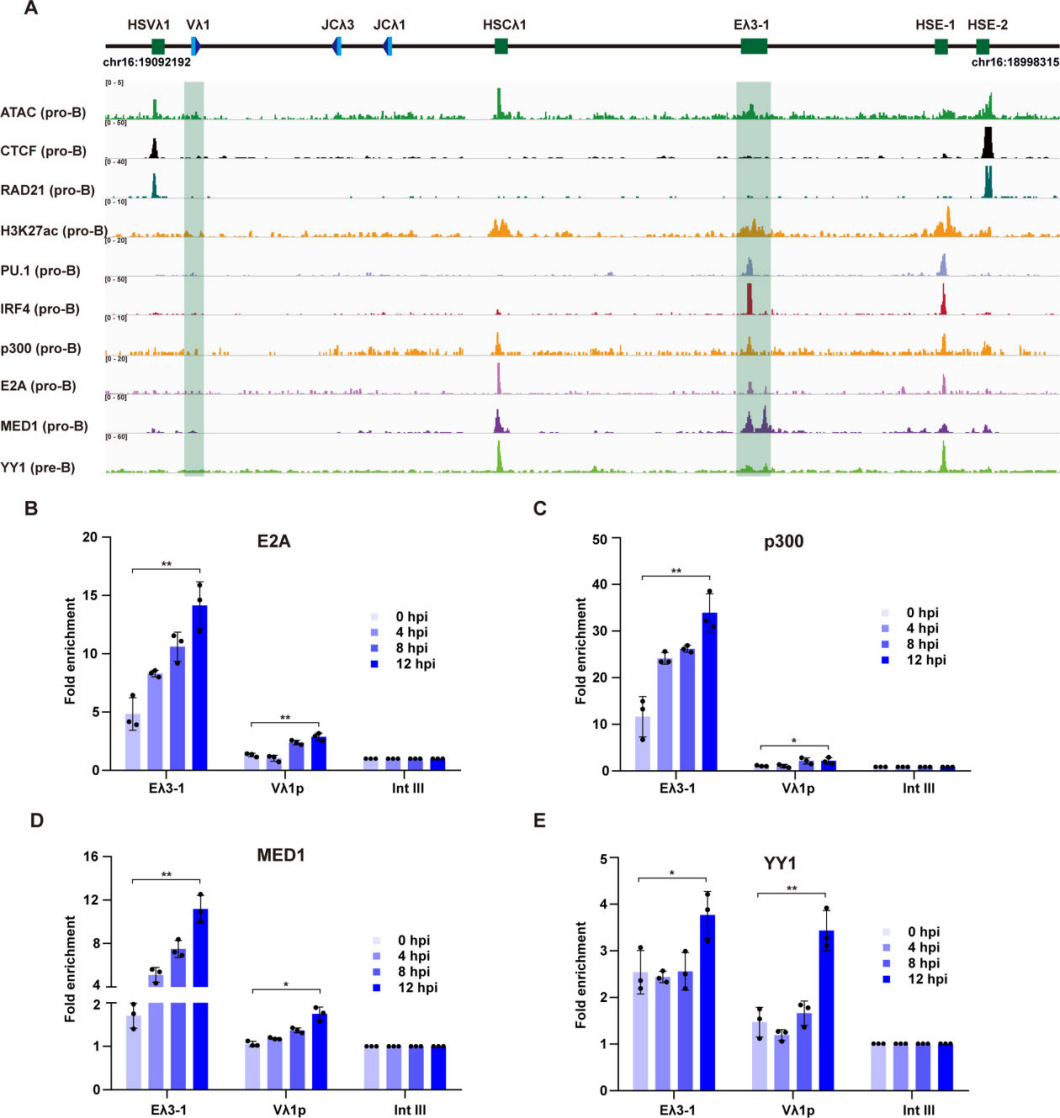
IRF4 triggers sequential recruitment of diverse transcription factors. (**A**) ATAC-seq and ChIP-seq data of architecture factors (CTCF, RAD21 and YY1), the enhancer mark (H3K27Ac) and transcription activators (PU.1, IRF4, E2A and MED1) mapped to the 3′ half of the Igλ locus. All data are from pro-B cells except YY1, which is from pre-B cells. (**B–E**) E2A, p300, MED1 and YY1 binding at Eλ3–1 and Vλ1p were analysed by ChIP-qPCR in PIPER-15 cells following induction. The fold enrichment at Eλ3–1, Vλ1p and Intgene III (negative control region) is shown. All values are normalized to binding at Intgene III as a negative control. Error bars show standard error of the mean (SEM) from three biological replicates.

Enhancer-mediated activation requires the coordinated action of multiple transcription factors, including histone modifying enzymes, lineage-specific transcription factors and architecture factors (5). To identify the proteins involved in Eλ3–1-mediated activation, published ChIP-seq data from primary pro-B cells and pro-B- derived cell lines (pre-B for YY1) were analysed. In addition to IRF4 and PU.1, significant enrichment of E2A, p300, Mediator and YY1 is observed at the Eλ3–1 enhancer (Figure 3A). Although activator binding is detected in pro-B cells, this may be explained by low levels of Igλ transcription in these cells, which is increased 8-fold upon transition to pre-B cells (7,45). To determine which factors play key roles in the sharp, coordinate increase in Vλ1 and Jλ1 transcription, we capitalised on the inducible nature of PIPER-15 cells to systematically analyse the temporal recruitment of each factor.

The basic helix-loop-helix (bHLH) transcription factor E2A interacts with IRF4 (19) and knockout studies showed that it is crucial to promote non-coding transcription of unrearranged Igλ gene segments in pre-B cells (46). Consistent with this, ChIP-Seq data show substantial E2A binding at the Eλ3–1 enhancer (Figure 3A) and ChIP-qPCR detects a significant increase in E2A at both Eλ3–1 and Vλ1p from pro- to pre-B cells (Supplementary Figure S3A). Complementary temporal ChIP analyses in PIPER-15 cells suggest that E2A is enriched at Eλ3–1 prior to induction and that binding increases gradually following IRF4 binding (Figure 3B). A similar temporal change is observed at the Vλ1 promoter although here, E2A binding is much lower (Figure 3B).

p300 is a histone acetyltransferase that exerts its function in concert with numerous transcription factors and mediates acetylation of histones close to enhancers and promoters to generate more flexible and accessible chromatin (47). Co-immunoprecipitation experiments demonstrated that E2A directly interacts with several histone acetyltransferases, including p300, that act in synergy with p300 to activate the Igκ locus (48,49). Similar to E2A, there is a peak of p300 binding at Eλ3–1 (Figure 3A) in primary pro-B cells, which is significantly increased in pre-B cells (Supplementary Figure S3A). Temporal ChIP analysis in PIPER-15 cells shows that the largest increase of p300 binding at Eλ3–1 is from 0 to 4 hpi, followed by a more gradual increase to 12 hpi (Figure 3C). A moderate, but reproducible, increase of binding is also observed at the Vλ1 promoter (Figure 3C). Consistent with an increase in p300 activity at the enhancer and promoter, ATAC-seq and H3K27ac ChIP-seq data show increased chromatin acetylation/accessibility at Eλ3–1 and its target promoters from primary pro- and pre-B cells (Supplementary Figure S3B). These changes in E2A and p300 binding therefore likely contribute to Vλ1 activation. However, neither shows the binding kinetics consistent with the sharp increase in Vλ1 transcription from 8–12 hpi and neither is known to stabilise enhancer–promoter interactions.

The Mediator complex, however, is an evolutionarily conserved, multi-subunit protein complex that plays an essential role in enhancer–promoter communications (50). This complex consists of more than 30 subunits which are organized into four distinct modules: the head, middle, tail and kinase modules (51). The head and middle modules interact with RNAPII and other components of the preinitiation complex (52,53) whereas tail module subunits physically interact with enhancer-bound transcription activators (54). Thus, it was suggested that Mediator provides a physical bridge between transcription activators at enhancers and the preinitiation complex at promoters (50), a role supported by recent short term knock-down studies and high resolution analysis of long range interactions (55). Previous co-immunoprecipitation assays revealed that IRF4 directly interacts with MED23 (56), which is the largest subunit in the tail module and is essential for early B cell development (57). To determine if MED23 is required for Igλ activation, shRNA-mediated knockdown was performed, resulting in a dramatic reduction in MED23 protein levels in cells expressing shRNA against MED23 (shMED23) compared to scrambled shRNA (shSCR; Supplementary Figure S3C, left). Crucially, Vλ1 transcription is also significantly decreased in shMED23 PIPER-15 cells (Supplementary Figure S3C) as is the interaction between Eλ3–1 and the Vλ1 and Jλ1 promoters following induction (Supplementary Figure S3C, right). These data therefore suggest that MED23 is essential for the coordinate activation of Vλ1 and Jλ1 transcription.

Ideally, the role of MED23 would be further investigated via temporal ChIP but ChIP-grade anti-MED23 antibodies are not available. Such antibodies are available, however, against MED1, the largest subunit of the Mediator complex, located in the middle module. Since this is part of the functional core of Mediator (58), analysis of MED1 binding is expected to mirror that of MED23. To first verify that MED1 is required for Vλ1 transcription, its expression was knocked down: Western blotting confirmed that MED1 protein levels are dramatically decreased (Supplementary Figure S3D), correlating with significantly reduced Vλ1 transcription (Supplementary Figure S3D); crucially, however, transcription of *Irf4*, *Ctcf* and *Smc1a* is not significantly altered in either MED1 or MED23 knock-down cells (Supplementary Figure S3E) suggesting loss of MED1/23 does not uniformly decrease transcription. Next, ChIP analysis was used to investigate how Mediator contributes to Vλ1 activation. MED1 binding to Eλ3–1 and Vλ1 increases significantly from pro- to pre-B cells (Supplementary Figure S3A) as well as in PIPER-15 cells following IRF4 induction. Here, the biggest relative increase at Eλ3–1 is from 0 to 4 hpi but further gradual increases are observed to 12 hpi (Figure 3D). Compared to the enhancer, MED1 binding to the Vλ1 promoter is low but reproducible and correlates with Vλ1 transcription, albeit without the sharp increase between 8 and 12 hpi (Figure 3D). Together, these data suggest that Mediator recruitment by IRF4 is vital for Igλ transcription and may contribute to enhancer/promoter bridging.

YY1 is a ubiquitously expressed, zinc finger DNA binding protein that activates or represses transcription, depending on the context in which it binds (59). YY1 plays an important role in chromatin folding of the IgH locus, where a YY1 conditional knockout led to decreased chromatin looping (16). Published ChIP-seq data indicate that YY1 is also enriched at the Eλ3–1 enhancer in pre-B cells (Figure 3A). To investigate if YY1 influences Vλ1 transcription and/or Igλ chromatin organization, shRNA against YY1 was expressed in PIPER-15 cells. Both YY1 protein levels (Supplementary Figure S3F) and Vλ1 transcription were dramatically reduced (Supplementary Figure S3F), as is the Eλ3–1 interaction frequency with Vλ1 (and Jλ1) following induction, as determined by 3C (Supplementary Figure S3F). Control experiments confirmed that transcription of *Irf4*, *Ctcf* and *Smc1a* are relatively unchanged (Supplementary Figure S3G). We find further that YY1 binding to Eλ3–1 and Vλ1 is significantly increased from pro- to pre-B cells (Supplementary Figure S3A). These data therefore imply that YY1 is essential for Vλ1 transcription activation and chromatin organization of the Igλ locus. To determine at which stage YY1 is required, temporal ChIP analysis was carried out following IRF4 induction. Intriguingly, YY1 is enriched at both the Eλ3–1 enhancer and Vλ1 promoter but its binding only increases significantly from 8 to 12 hpi at both regions (Figure 3E). This therefore correlates very well with the sharp increase in Vλ1 and Jλ1 transcription and suggests that YY1 is pivotal to this increase.

To better understand how RNAPII is recruited to achieve this increased transcription, ChIP experiments with antibodies against serine-5 and serine-2 phosphorylated C-terminal domain were carried out. RNAPII is already present at the Eλ3–1 enhancer and Vλ1 promoter at low levels prior to IRF4 induction, consistent with low levels of Vλ1 transcription in pro-B cells (7). Upon induction, both serine-5 and serine-2 phosphorylated RNAPII gradually increase at the Eλ3–1 enhancer (Supplementary Figures S4A and S4B), concomitant with enhancer activation and correlating with Mediator binding to the enhancer (Figure 3D). At the Vλ1 promoter, an increase in serine-5 phosphorylated RNAPII is observed at 8 hpi, corresponding to increased 3C interactions between Eλ3–1 and Vλ1 (Figure 2E). Binding of serine-5 phosphorylated RNAPII then decreases concomitant with a significant increase of promoter-bound serine-2 phosphorylated RNAPII between 8 and 12 hpi. These data therefore suggest that RNAPII is initially recruited to the enhancer and transferred to the promoter via enhancer–promoter interactions during transcriptional activation. It is also notable that the sharp increase in serine-2 phosphorylated RNAPII at the Vλ1 promoter correlates with increased YY1 binding and Vλ1 transcription.

### IRF4 mediated formation of an enhancer hub is essential for Igλ activation

Whilst the above analyses identify which activators play important roles in Vλ1 activation, they do not explain how Jλ1 is coordinately upregulated, nor do they show if other gene regulatory elements are required. Antigen receptor loci typically contain multiple gene segments and corresponding regulatory DNA elements that can span mega-base sized chromatin regions. To characterize additional *cis-*acting elements, published ATAC-seq and ChIP-seq data from pro-B cells were reprocessed and mapped to the murine Igλ locus (Figure 3A). In addition to Eλ3–1, four further regions of open chromatin were found in the 3′ domain of Igλ, namely HSCVλ1, HSCλ1, HSE-1 and HSE-2 in primary pro-B cells (Figure 3A) and PIPER-15 cells (Supplementary Figure S4C).

**Figure 4.**
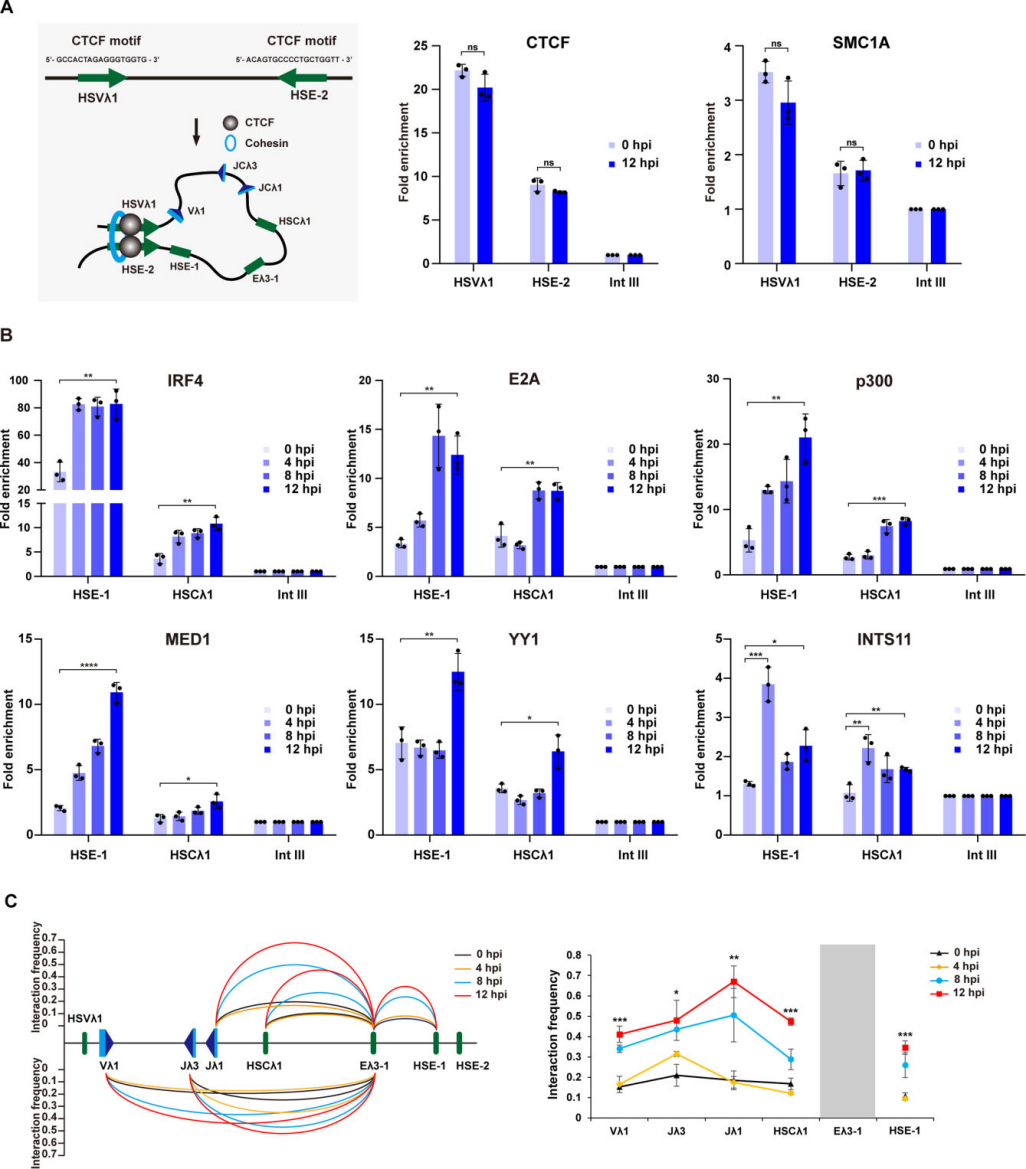
IRF4-mediated formation of an enhancer hub activates the Igλ locus. (**A**) Left, upper: CTCF motifs at HSE-2 and HSVλ1 lie in a convergent orientation (green arrows). Left, lower: Schematic showing formation of the chromatin loop that brings HSE-1 and the Vλ1 promoter into close proximity. Right: CTCF and SMC1A (cohesin subunit) binding to HSE-2 and HSVλ1 measured by ChIP-qPCR in PIPER-15 cells. The fold enrichment at HSE-2, HSVλ1 and Intgene III (negative control region) is shown. (**B**) Binding of IRF4, E2A, p300, MED1, YY1 and INTS11 to HSE-1 and HSCλ1 analysed by ChIP-qPCR in PIPER-15 cells following induction. The fold enrichment at HSE-1 and HSCλ1 is shown. All values are normalized to binding at Intgene III as a negative control. (**C**) Temporal 3C analysis of chromatin interactions in the 3′ half of the Igλ locus. Analysis of the relative interaction frequency of Dpn II fragments from the Eλ3–1 viewpoint in PIPER-15 cells at 0, 4, 8 and 12 hpi. Data were normalized using an interaction within the *Ercc3* locus and are the average of three experimental repeats (Supplementary Table 1). The plots to the right of the same data show the significance of the difference in interactions between 0 and 12 hpi. Error bars show standard error of the mean (SEM) from three biological replicates.

Intriguingly, two of these sites, HSVλ1 and HSE-2, lie at the very 5′ and 3′ of the 3′ domain and show peaks of CTCF and cohesin (RAD21) binding. These essential architecture factors generate chromatin loops that separate the genome into diverse domains and thus may create an insulated neighbourhood domain in the 3′ region of the Igλ locus (Figure 4A). Consistent with this idea, CTCF typically mediates chromatin loops between convergent CTCF motifs (60), which is the orientation observed at HSVλ1 and HSE-2 (Figure 4A). Not only this, but Hi-C data from pre-B cells indicate substantial interactions between HSVλ1 and HSE-2 (Figure 1C) and ChIP-qPCR experiments show a marked enrichment of CTCF and SMC1A (cohesin subunit) at HSVλ1 and HSE-2 that is unaltered in PIPER-15 cells following IRF4 induction (Figure 4A) and between primary pro- and pre-B cells (Supplementary Figure S4D). These data therefore imply that CTCF/cohesin connects HSVλ1 and HSE-2 via a chromatin loop to create the Igλ locus 3′ domain. Notably, this loop brings HSE-1 and the Vλ1 promoter into closer proximity, which may facilitate Vλ1 activation (Figure 4A, lower).

We next examined which elements might cooperate within the large CTCF/cohesin-generated loop to orchestrate Igλ locus activation. Similar to Eλ3–1, both HSCλ1 and HSE-1 are open chromatin regions with a high level of H3K27ac and p300 binding (Figure 3A) and thus display the characteristics of active enhancers. Consistent with this idea, ChIP-seq data from pro-B cells shows transcription factor binding peaks at HSE-1 and HSCλ1 that are very similar to those at Eλ3–1 (Figure 3A). Moreover, the IRF4 ChIP-qPCR signal is highly enriched at Eλ3–1 and HSE-1 (Figure 4B), possibly due to recruitment by pre-bound PU.1 (39,42) whereas low levels of IRF4 are present at HSCλ1 where PU.1 is absent. These data therefore imply that the newly identified enhancer-like elements HSE-1 and HSCλ1 play an integral role in Igλ locus activation. In support of this, significant interactions among these three enhancer elements are seen in Hi-C data (Figure 1C), suggesting that they may form an enhancer hub.

To test this idea, temporal 3C analysis was performed using Eλ3–1 as a viewpoint. Prior to Igλ activation, Eλ3–1 exhibits limited contacts with HSE-1 and HSCλ1 or with the unrearranged Vλ1, Jλ1 and Jλ3 gene segments. Following induction, chromatin contacts do not change dramatically by 4 hpi. Remarkably, however, a substantial increase in interaction frequency between Eλ3–1 and Vλ1, Jλ1 and Jλ3 occurs by 8 hpi, with a further increase by 12 hpi (Figure 4C), mirroring significant increases in transcription (Figure 2B). Not only this, but the interaction frequency between Eλ3–1 and HSE-1 as well as HSCλ1 correlates well with the changes in interactions between Eλ3–1 and Vλ1, Jλ1 and Jλ3. These data therefore suggest that Eλ3–1 interacts with HSCλ1 and HSE-1 to from an enhancer hub and that the target genes, Vλ1, Jλ1 and Jλ3, are concurrently brought into proximity of this hub, allowing their coordinate activation.

To further investigate the enhancer hub idea, we next separately knocked out the PU.1/IRF4 binding sites within the Eλ3–1 and HSE-1 enhancers using CRISPR/Cas9 (Supplementary Figure S4E and Supplementary Table 2). Changes in Vλ1 and Jλ1 transcription were then determined as well as alterations in enhancer–promoter interactions from the HSE-1 and Eλ3–1 viewpoints. Consistent with idea that IRF4 is central to locus activation, removal of its motif from either enhancer results in a significant reduction in both Vλ1 and Jλ1 transcription (Supplementary Figure S4F), that correlates with a dramatic loss of both enhancer-enhancer and enhancer–promoter interactions throughout the entire locus (Supplementary Figures S4G and S4H). The fact that loss of IRF4 binding to just one enhancer, either Eλ3–1 or HSE.1, causes such fundamental changes to the whole locus, supports the idea of coordinated enhancer hub formation.

The striking similarity of transcription factor motifs at Eλ3–1, HSCλ1 and HSE-1 suggests that they may share comparable dynamic transcription factor binding profiles that could facilitate enhancer hub formation. To investigate this, ChIP analyses of IRF4, E2A, p300, MED1 and YY1 were performed at Eλ3–1, HSE-1 and HSCλ1 in pro-B, pre-B and PIPER-15 cells. Temporal analyses showed that, similar to its recruitment to Eλ3–1, IRF4 binding to HSE-1 is an early event that reaches its maximal level at 4 hpi, (Figure 4B). IRF4 binding to HSCλ1 shows a similar temporal pattern of recruitment, although here, in the absence of PU.1 (Figure 3A), binding occurs at only low levels (Figure 4B). Just as for Eλ3–1, binding to HSE-1 and HSCλ1 is also significantly increased from pro-B to pre-B cells (Supplementary Figure S5).

E2A and p300 binding to HSE-1 and HSCλ1 also show a similar temporal pattern of recruitment to that seen at Eλ3–1, with significantly increased binding at 8 and 12 hpi (Figure 4B), which is also consistent with data from pro- and pre-B cells (Supplementary Figure S5). Together, these data suggest that IRF4 interacts directly with Eλ3–1, HSE-1 and HSCλ1 and this increased IRF4 binding results in recruitment of E2A and p300 to generate open chromatin regions.

**Figure 5.**
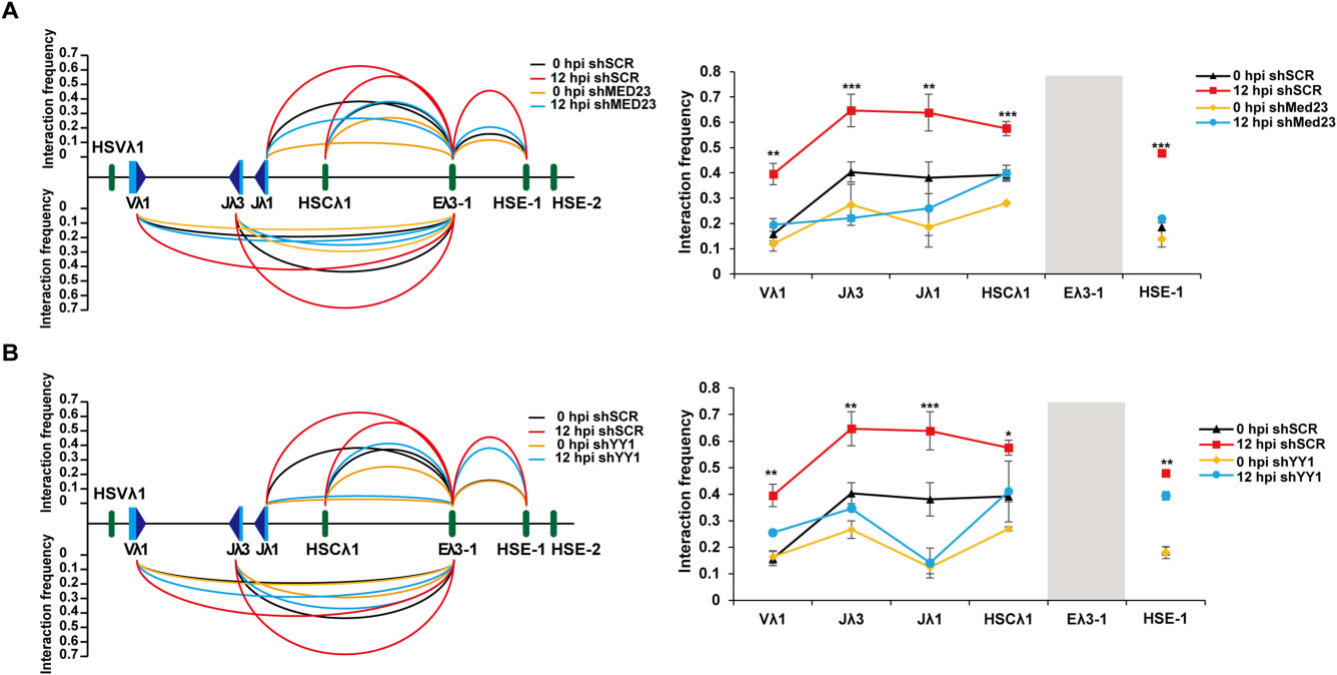
MED23 and YY1 are essential for Igλ locus contraction. (**A**) Analysis of the relative interaction frequency of Dpn II fragments from the Eλ3–1 viewpoint in PIPER-15 cells expressing an shRNA targeting *Med23*. The height of curves between Eλ3–1 and other genomic fragments represents the average value of interaction frequency obtained from three experimental repeats (Supplementary Table 1). Data were normalized using an interaction within the *Ercc3* locus. The plots to the right show the significance of the difference in interactions at 12 hpi between shSCR and shMED23. (**B**) Analysis of the relative interaction frequency of Dpn II fragments from the Eλ3–1 viewpoint in PIPER-15 cells expressing an shRNA targeting *Yy1*. The height of curves between Eλ3–1 and other genomic fragments represents the average value of interaction frequency obtained from three experimental repeats (Supplementary Table 1). Data were normalized using an interaction within the *Ercc3* locus. The plots to the right show the significance of the difference in interactions at 12 hpi between shSCR and shYY1. Notably, the biggest increase in locus interactions is between 4 and 8 hpi (Figure 4C) whereas the biggest increase in YY1 binding is between 8 and 12 hpi. However, considerable YY1 binding is observed prior to locus induction; this could stabilise long range interactions as they are formed and may explain why knockdown of YY1 impacts so significantly on locus folding.

Formation of the enhancer hub requires the constituent enhancers to be brought into closer proximity. To determine if Mediator is involved, published MED1 ChIP-seq from pro-B cells was analysed. As can be seen in Figure 3A, MED1 is already present at HSE-1 and HSCλ1 at low levels; likewise, low levels of IRF4 are found at both elements consistent with low level locus activity in pro-B cells and it is possible Mediator is recruited through direct interactions with IRF4. Following induction of PIPER-15 cells, a gradual increase in MED1 binding to HSE-1 and HSCλ1 is observed (Figure 4B), mirroring its binding to Eλ3–1 (Figure 3D), and consistent with the significantly increased binding between pro- and pre-B cells (Supplementary Figure S5). To determine if Mediator is essential to establish interactions that lead to enhancer hub formation, 3C analysis was performed in MED23 knock-down PIPER-15 cells, with and without IRF4 induction: Eλ3–1, HSE-1 and HSCλ1 (Figure 5A) contacts are dramatically decreased, as are interactions between Eλ3–1 and the Jλ1, Vλ1 and Jλ3, gene segments (Figure 5A). These data therefore imply that Mediator is vital for IRF4-mediated formation of the Igλ enhancer hub and for interactions with gene segment promoters, leading to their coordinate activation.

It is notable that knockdown of MED1 (and YY1) reduces the interactions seen at 0 hpi compared to those seen with the scrambled RNA (orange with black plots, respectively). This further correlates with reduced Vλ1 transcription at 0 hpi in the presence of shMED1/23 versus shSCR (and shYY1 versus shSCR; Supplementary Figure S3C, D, F). This may be because knock-down of these factors causes loss of the low level (IRF4-dependent) activity of the Igλ locus in pro-B cells (7).

To measure the impact of YY1 on enhancer hub formation, temporal analysis of YY1 binding was performed in PIPER-15 cells. As can be seen in Figure 4B, YY1 occupancy at HSE-1 and HSCλ1 dramatically increases from 8 hpi to 12 hpi, mirroring its binding to Eλ3–1 (Figure 3E) and the large increase in Vλ1 and Jλ1 transcription following induction (Figure 2B). Significantly increased YY1 enrichment at these two enhancers is also observed in pre-B compared to pro-B cells (Supplementary Figure S5) and notably, the fold-change in binding from pro- to pre-B cells as well as from 8 to 12 hpi in PIPER-15 cells is very similar at all enhancer-like elements, including Eλ3–1. To determine if YY1 binding also modulates locus folding, its expression was depleted in PIPER-15 cells (Supplementary Figure S3F). Remarkably, this resulted in a significant reduction of the enhanced interactions at 12 hpi between Eλ3–1 and HSCλ1 (Figure 5B and Supplementary Figure S3F) as well as between Eλ3–1 and its target genes Vλ1, Jλ1 and Jλ3 (Figure 5B), correlating with diminished Vλ1 transcription (Supplementary Figure S3F). Furthermore, knockout of the YY1 site in HSCλ1 almost eliminated Eλ3–1/HSCλ1 interactions as well as Eλ3–1 interactions with Vλ1, Jλ1 and Jλ1, and resulted in significantly reduced Vλ1 transcription (61). YY1 therefore appears to be pivotal to the interactions between the enhancers and target genes and the coordinate activation of otherwise distant Vλ1, Jλ1 and Jλ3 promoters, although not Eλ3–1/HSE.1 interactions. Temporal analyses suggest that YY1 functions later than Mediator, perhaps by stabilising pre-formed interactions; nonetheless, the dramatic disruption of locus folding in the absence of YY1, implies that its function is vital.

### Antisense eRNAs encoded by Eλ3-1 repress YY1 recruitment

YY1 has a relatively low affinity for DNA (62) and although its binding may be stabilised via IRF4-interacting proteins, such as p300 (63), exactly how YY1 binding is stabilised, is unclear. Given its vital role in locus folding, the mechanism of YY1 stabilisation could be pivotal to locus activation. Enhancer RNAs (eRNAs) are a sub-class of non-coding RNAs that are transcribed from active enhancers and have been demonstrated to be involved in enhancer–promoter loop formation and target gene activation (64). Previous publications demonstrated that eRNAs can interact with diverse transcription factors, including cohesin (65), Mediator (66), YY1 (67) and p300 (68). Notably, RNA-seq reads map to Eλ3–1 (Supplementary Figure S6A) and Eλ3–1 eRNA expression levels increase significantly from primary pro-B to pre-B cells (Supplementary Figure S6B), suggesting that they may be important to Igλ locus activation.

**Figure 6.**
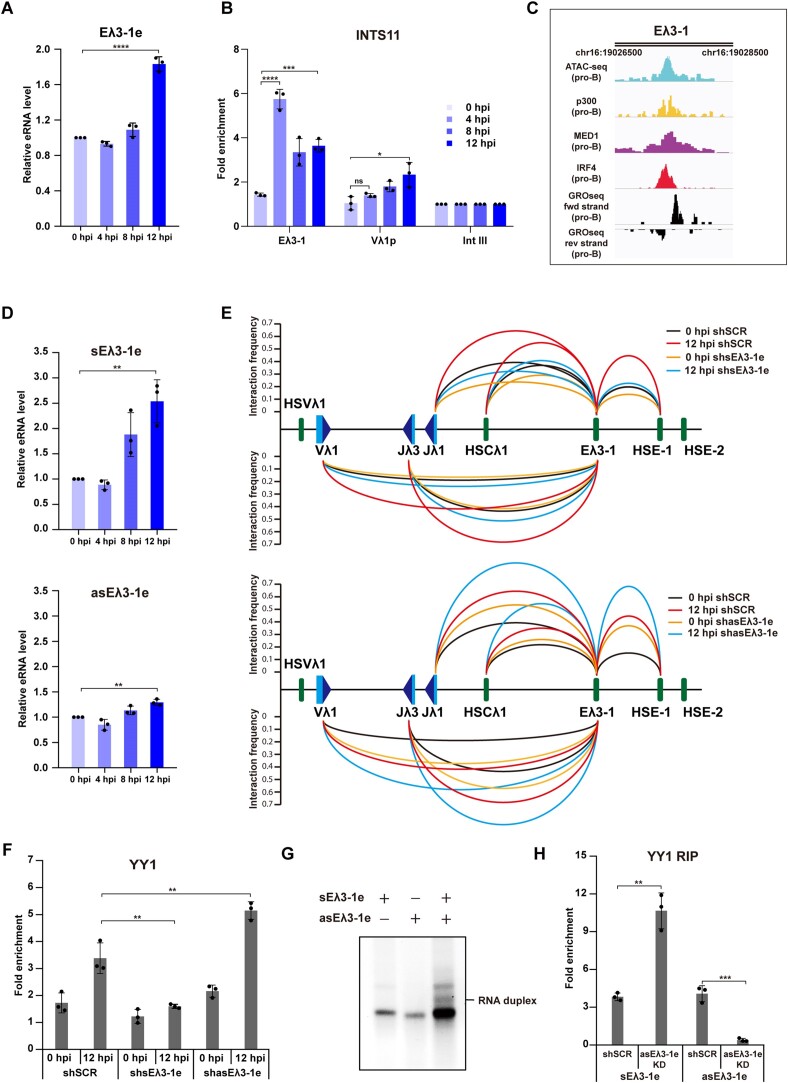
Antisense eRNAs encoded by Eλ3–1 repress YY1 recruitment. (**A**) Temporal analysis of Eλ3–1 transcription in PIPER-15 cells. Transcription of the Eλ3–1 enhancer was analysed by RT-qPCR in PIPER-15 cells following induction. Data are normalized to *Hprt* expression. (**B**) Integrator is recruited to both Eλ3–1 and Vλ1p in PIPER-15 cells. Integrator binding at the Eλ3–1 enhancer and Vλ1 promoter analysed by ChIP-qPCR in PIPER-15 cells following induction. The fold enrichment at Eλ3–1, Vλ1p and Intgene III is shown. Binding falls from peaks levels at 4 hpi but remains above that at 0 hpi; this may be due to Integrator turning over stalled RNAPII (86), that is transferred to the promoter at later time points. All values are normalized to binding at Intgene III as a negative control. (**C**) Left: GRO-seq data from pro-B cells was reanalysed using the Galaxy web server. Signal peaks of ATAC-seq and ChIP-seq data from pro-B cells map to the central region of the Eλ3–1 enhancer. Visualization of the mapped reads was performed in IGV. Genomic coordinates of the Eλ3–1 enhancer are shown. (**D**) Temporal analysis of expression of sense (sEλ3–1e; upper) and antisense (asEλ3–1e; lower) eRNAs by RT-qPCR in PIPER-15 cells following induction. Data are normalized to *Hprt* expression. (**E**) Analysis of the relative interaction frequency of Dpn II fragments from the Eλ3–1 enhancer in PIPER-15 cells expressing scrambled (shSCR), or shRNAs against sense (shsEλ3–1e, upper) or antisense (shasEλ3–1e, lower) eRNAs. The significance of the difference in interactions is given in Supplementary Figures S6H and S6I. (**F**) YY1 binding to Eλ3–1 in PIPER-15 cells expressing scrambled (shSCR), sense shsEλ3–1e and antisense shasEλ3–1e Eλ3–1 eRNAs. All values are normalized to binding at Intgene III as a negative control. (**G**) Native agarose gel electrophoresis of sense (sEλ3–1e) and antisense (asEλ3–1e) eRNAs. H. YY1 binding to sense (sEλ3–1e) and antisense (asEλ3–1e) eRNAs in PIPER-15 cells expressing scrambled (shSCR) or shRNA against antisense (shasEλ3–1e) eRNA. The fold enrichment over input is shown. Error bars show standard error of the mean (SEM) from three biological replicates.

To further investigate this, eRNA expression was analysed temporally via RT-qPCR following IRF4 induction. As can be seen in Figure 6A, total Eλ3–1 eRNA levels show a marginal increase between 4 and 8 hpi, prior to increased YY1 binding at Eλ3–1 (Figure 3E). A large increase is observed however, between 8 and 12 h which correlates well with the largest increase in YY1 binding (Figure 3E). YY1 was previously demonstrated to be trapped by RNAs tethered at enhancers (67) and these data suggest that increased YY1 binding at Eλ3–1 may be facilitated by eRNAs.

Similar to mRNAs, eRNAs are also transcribed by the RNAPII machinery. However, their 3′ ends are processed by the Integrator complex which facilitates eRNA maturation and their release from transcribing RNAPII (69). Consistent with a role for eRNA, temporal ChIP analysis of Integrator (the INTS11 subunit) binding in PIPER-15 cells shows that it reaches its highest level at Eλ3–1, HSE-1 and HSCλ1 at 4 hpi (Figures 4B and 6B), just prior to increased eRNA levels. Increases in Integrator occupancy are also observed at all three enhancer-like elements and the Vλ1 promoter from pro- to pre-B cells (Supplementary Figures S5 and S6C).

Genome-wide analysis suggests that the majority of enhancers are transcribed bidirectionally (70) and GRO-seq data from pro-B cells identifies a number of reads that map to both sense and antisense strands of the Eλ3–1 enhancer as well as HSCλ1 and HSE-1 (Figures 6C and S6D). Temporal analysis of sense and antisense Eλ3–1 eRNA expression, following reverse transcription with strand-specific primers, shows that sense Eλ3–1 eRNA starts to increase between 4 and 8 hpi (Figure 6D), whereas changes in anti-sense eRNA are much lower than sense eRNA. Consequently, the relative amount of sense eRNA increases compared to antisense.

To determine the impact of these eRNAs on Vλ1 transcription, sense and antisense Eλ3–1 eRNAs were separately knocked down in PIPER-15 cells. Sense Eλ3–1 eRNA is dramatically reduced in PIPER-15 cells expressing the relevant shRNA (Supplementary Figure S6E). Likewise, antisense Eλ3–1 eRNA is reduced by more than 70% (Supplementary Figure S6F). Similar to the activation by eRNAs at other loci (71), knockdown of sense Eλ3–1 eRNA (shsEλ3–1e) results in significantly decreased Vλ1 transcription (Supplementary Figure S6E). Remarkably, however, compared to shSCR PIPER-15 cells, Vλ1 transcription increases significantly in antisense Eλ3–1 eRNA knock-down (shasEλ3–1e) cells (Supplementary Figure S6F). Consistent with this, overexpression of antisense Eλ3–1 eRNA dramatically reduces Vλ1 transcription (Supplementary Figure S6G).

Notably, the level of Vλ1 transcription is altered in the presence of sense or antisense shRNA at 0 hpi, in a similar way to at 12 hpi, when compared to the respective scrambled control. This may be because the Igλ locus is already active at low levels at 0 hpi (7) and knockdown of the eRNAs likely affects both the basal (pro-B-like), as well as induced Vλ1 transcription. Consequently, the fold-induction (comparing 0 to 12 hpi) appears similar between the scrambled shRNA control and the sense or antisense eRNA knockdown. Given that the knockdown affects both uninduced and induced transcription, we compare scrambled and knock-down shRNA levels at either 0 or 12 hpi (Supplementary Figures S6E and S6F).

Previous publications showed that eRNAs are essential to establish enhancer–promoter interactions (65,72). Therefore, to investigate the impact of sense and antisense Eλ3–1 eRNAs on formation of the Vλ1-Eλ3–1 chromatin loop, 3C analysis of Eλ3–1 to Vλ1 interactions was performed in shsEλ3–1e and shasEλ3–1e PIPER-15 cells, respectively. Consistent with the observed transcription changes, Vλ1-Eλ3–1 interactions are significantly reduced in shsEλ3–1e PIPER-15 cells at 12 hpi (Figure 6E and Supplementary Figure S6H), indicating that sense Eλ3–1 eRNA is vital to establish enhancer–promoter chromatin loops. However, remarkably, Eλ3–1 to Vλ1 interaction frequency is significantly increased in shasEλ3–1e PIPER-15 cells (Figure 6E, lower and Supplementary Figure S6I), implying that antisense eRNAs repress enhancer/promoter loop formation and reduce target gene transcription.

Enhancer RNAs are known to exert their functions by interacting with diverse transcription factors (66–68). Temporal ChIP analysis showed that Eλ3–1 eRNAs increase just prior to increased YY1 enrichment at Eλ3–1, suggesting that expression of eRNAs may be a prerequisite for stable YY1 binding; conversely, diminished locus folding in eRNA knock-down cells may be caused by reduced YY1 binding. To test this idea, ChIP-qPCR analysis of YY1 binding to Eλ3–1 was performed in the shsEλ3–1e and shasEλ3–1e PIPER-15 cells. As can be seen in Figure 6F, knockdown of the sense Eλ3–1 eRNA leads to decreased YY1 occupancy at Eλ3–1, suggesting that eRNA-mediated chromatin folding is indeed associated with YY1 binding. Intriguingly, however, YY1 binding to Eλ3–1 is increased in the antisense Eλ3–1 eRNA knock-down cells (Figure 6F), indicating that the antisense eRNAs repress YY1 recruitment.

The bidirectional sense and antisense Eλ3–1 eRNAs appear to arise from different regions of the Eλ3–1 enhancer (Figure 6C) but short regions of homology (Supplementary Figure S6J) are present. It therefore seemed possible that antisense eRNAs interact with sense eRNAs to regulate YY1 recruitment to enhancers. To test this, sense and antisense Eλ3–1 eRNA were generated via *in vitro* transcription and RNA-RNA hybridization experiments performed. Interactions between antisense and sense Eλ3–1 eRNAs are indeed observed, evidenced by duplex formation *in vitro* (Figure 6G); by contrast, control experiments using a random RNA that lacks sequence homology, failed to hybridise (Supplementary Figure S6K). To further investigate the role of antisense enhancer *in vivo*, RNA immunoprecipitation was performed; this showed that YY1 pulls down ∼2.5-fold more sense eRNAs in the antisense eRNA knock-down cells compared to PIPER-15 cells (Figure 6H). Together, these data indicate that antisense eRNAs interact with sense eRNAs to suppress YY1 recruitment and stable locus activation.

## DISCUSSION

Enhancer-mediated activation is vital for the correct levels of transcription at the right developmental stages. Whilst numerous studies have shown that enhancers trigger activation by physically interacting with their cognate promoters, antigen receptor loci pose a unique problem in that non-coding transcription must be coordinately upregulated through at least two distant, complementary gene segments prior to their recombination. Here, we capitalised on the finding that increased levels of just a single transcription factor, IRF4, is sufficient to completely activate the murine Igλ locus, to develop a system in which Igλ gene transcription can be reliably induced. Using this novel inducible system, we confirm for the first time, that non-coding transcription through V and J gene segments is indeed coordinately upregulated. We then systematically analysed the temporal recruitment of transcription activators, as well as long range chromatin folding and find that three enhancer elements in the 3′ domain of the Igλ locus show remarkably similar dynamics of activator binding. Our temporal analyses show further that these enhancers are brought together into an activating hub, concomitant with the recruitment of V and J promoters and their transcription activation. Co-localisation of enhancers and promoters within the same activating hub is thus central to coordinate Vλ1 and Jλ1 activation. Given that other antigen receptor loci also undergo locus folding, it is highly feasible that similar mechanisms are adopted to coordinately activate their complementary gene segments, prior to recombination (73–75).

Our studies show further that establishment of the functional enhancer–promoter hub requires diverse transcription factors, including general transcription factors, lineage-specific transcription factors, histone modifiers, architecture transcription factors as well as eRNAs. By following transcription activation temporally, we could deduce which events correlate most closely with both locus folding and transcription upregulation and thus are potentially key regulatory steps. A sharp increase in Vλ1 and Jλ1 transcription is observed between 8 and 12 hpi that correlates extremely well with YY1 binding to all three enhancers, suggesting that this is a key event. YY1 functions as a transcription factor, but can also help to establish chromatin loops, especially enhancer–promoter loops (76). Consistent with the latter role, knockdown of YY1 reduces Vλ1 non-coding transcription and results in a severe disruption of long range chromatin interactions, without a significant loss of transcription at other loci. These data therefore suggest that YY1 binding is important to stabilise the active chromatin hub and to thereby achieve consistent transcription activation.

Our knock-down studies demonstrate that, in addition to YY1, Mediator is also vital for Vλ1 transcription and Igλ locus folding. Remarkably, long range interactions are altered very similarly upon loss of either transcription factor, suggesting that they interact with very similar regions, but function independently. Notably, enhancer–promoter, as well as enhancer-enhancer interactions are disrupted, which supports the idea that an active hub is formed between enhancer- and promoter-bound transcription factors. Given that there is an increase in long range interactions between 4 and 8 hpi without a change in YY1 binding, it appears that Mediator may establish enhancer/promoter interactions that are then stabilised by YY1.

The bridging role of Mediator between enhancers and promoters, although debated, has recently been shown to be important at 20 loci regulated by super-enhancers (55), and can explain how Mediator establishes the long range interactions (50). YY1, however, has both DNA and RNA binding activity as well as an intrinsically disordered domain that is distinct from its DNA binding domain (77). Consensus YY1 DNA binding motifs are not present at the Vλ1 nor Jλ1/3 promoters and whilst we find eRNA likely stabilises YY1 binding at Eλ3–1, its recruitment to promoters is unclear. Given the formation of an active hub, YY1 localisation to promoters may involve interactions between intrinsically disordered domains in both YY1 and other transcription activators in the enhancer/promoter hub (78).

Notably, knockout of either Eλ3–1 or HSE-1 enhancer results in a substantial reduction in Vλ1 and Jλ1 transcription and a significant reduction in most long range interactions. The requirement for both enhancers to achieve transcription activation may be explained if activation depends on threshold levels of activation potential within the enhancer–promoter hub. Although temporal analyses show that some transcription activation occurs prior to complete binding of all transcription factors, transcription levels are only modest and full transcription activation is only achieved upon increased YY1 binding, consistent with a vital role for YY1 in stabilising long range interactions.

Although YY1 interacts directly with Mediator (79) and p300 (63), these proteins bind to Eλ3–1 as early events in Igλ locus activation; YY1 binding, however, increases much later, suggesting that YY1 is recruited by other factors. YY1 contains RNA binding domains and previous studies demonstrated that YY1 can be recruited via enhancer-tethered eRNAs (67). Temporal analysis of the expression of eRNAs encoded by Eλ3–1 shows that changes in the level of eRNAs correlate very well with changes in YY1 binding: eRNAs start to increase from 4 hpi, just prior to increased YY1 binding, with their largest increase between 8 and 12 hpi, concomitant with the sharp increase in YY1 binding. It is also notable that serine-2 phosphorylated RNAPII at the Vλ1 promoter increases significantly between 8 and 12 hpi, correlating with the sharp increases in Vλ1 transcription, YY1 binding and sense eRNA. By contrast, binding of serine-5 phosphorylated RNAPII decreases from 8 to 12 hpi. Previous studies have demonstrated eRNA can recruit CDK9 of the P-TEFb complex (80) and it therefore seems possible that sense eRNA fulfils a second function of recruiting the P-TEFb complex to activate transcription.

Integrator is required for eRNA biosynthesis and its binding to Eλ3–1 reaches its highest level at 4 hpi, just prior to the increase of eRNAs. Consistent with concerted activity between activators, Integrator directly interacts with Mediator (81); given that Mediator binding to Eλ3–1 is an early event, it is feasible that Mediator recruits Integrator to modulate changes in eRNAs.

Most enhancers are transcribed bidirectionally but the function of the non-dominant eRNA is largely unknown. Our studies show that remarkably, the less dominant, antisense Eλ3–1 eRNA represses YY1 recruitment, raising the question of just how this is achieved. Tertiary structure is essential for eRNAs to recognize their binding partners. For example, 40% of sense eRNAs possess a functional eRNA regulatory motif (FERM) which can mediate interactions with diverse transcription activators, including YY1 (82). Although a FERM is not present in sense Eλ3–1 eRNA, other such motifs with specific tertiary structures may be present and antisense eRNA might disrupt such structures to block interactions with YY1. Alternatively, antisense eRNA might prevent other proteins such as p300, MED1, MED12 that interact with eRNAs (83–85), from tethering sense eRNA to enhancer elements. Consistent with the idea that antisense eRNA interacts with sense eRNA to regulate YY1 binding and/or eRNA tethering, *in vitro* RNA hybridization demonstrated sense/antisense eRNA interactions, despite each eRNA being encoded by distinct sequences. Consequently, the interaction dynamics between sense and antisense eRNAs may regulate transcription factor recruitment. Given that similar sense and antisense eRNAs are present at the other Igλ enhancers, it is possible that YY1 is recruited at tethered in similar way at HSE-1 and HSCλ1. Consistent with this idea, loss of one enhancer and its corresponding eRNA results in complete loss of Igλ chromatin folding.

Together our data show that Igλ locus activation requires the establishment of the correct chromatin environment that culminates in enhancers and promoters being brought into close proximity, triggering coordinated V and J activation. We demonstrated that HSE-2 and HSVλ1 establish a chromatin loop that seals the 3′ end of the Igλ locus and results in locus contraction, shortening the distance between Eλ3–1 and the unrearranged gene segments. CTCF/cohesin mediated folding, however, is unlikely to be sufficient to establish enhancer–promoter interactions. Instead, temporal 3C analysis showed that two other enhancer-like sequences in addition to Eλ3–1, namely HSE-1 and HSCλ1, have similar transcription factor binding dynamics and interact to form an enhancer hub. From this, we propose a three-step model to explain the chromatin structure changes during Igλ locus activation (Graphical abstract): Step 1: Formation of the CTCF/cohesin mediated chromatin loop between HSE-2 and HSVλ1; Step 2: IRF4 facilitates locus contraction through recruiting histone modifiers, Mediator and Integrator; Step 3: Upregulation of sense eRNAs causing recruitment of YY1 and stabilisation of Igλ folding. As a result of these chromatin changes, the unrearranged gene segments are brought into close proximity of the enhancer hub, establishing, in principle, how their coordinate activation is achieved.

## Supplementary Material

gkad741_Supplemental_Files

## Data Availability

The data underlying this article are available in the article and in its online supplementary material. The ATAC-seq data underlying this article are available under GEO accession GSE239925 and via the UCSC link: http://genome-euro.ucsc.edu/s/asmith151/2023%2D07%2D24%2DPIPER15%2D12hr%2DATAC%2Dseq.

## Notes

Present address: Alastair L. Smith, MRC Molecular Haematology Unit, MRC Weatherall Institute of Molecular Medicine, University of Oxford, Oxford OX3 9DS, UK.

Present address: Sarah L. Bevington, Labcorp Drug Development, Harrogate HG3 1PY, UK.
