# Supplementary material for: Temporal analyses reveal a pivotal role for sense and antisense enhancer RNAs in coordinate immunoglobulin lambda locus activation
Source: Nucleic Acids Res. 2023 Sep 13;51(19):10344–63. doi: 10.1093/nar/gkad741 (PMC10602925; doi:10.1093/nar/gkad741)
Supplement: gkad741_Supplemental_Files [file gkad741_supplemental_files.zip › Gao et al Supplementary data.pdf]

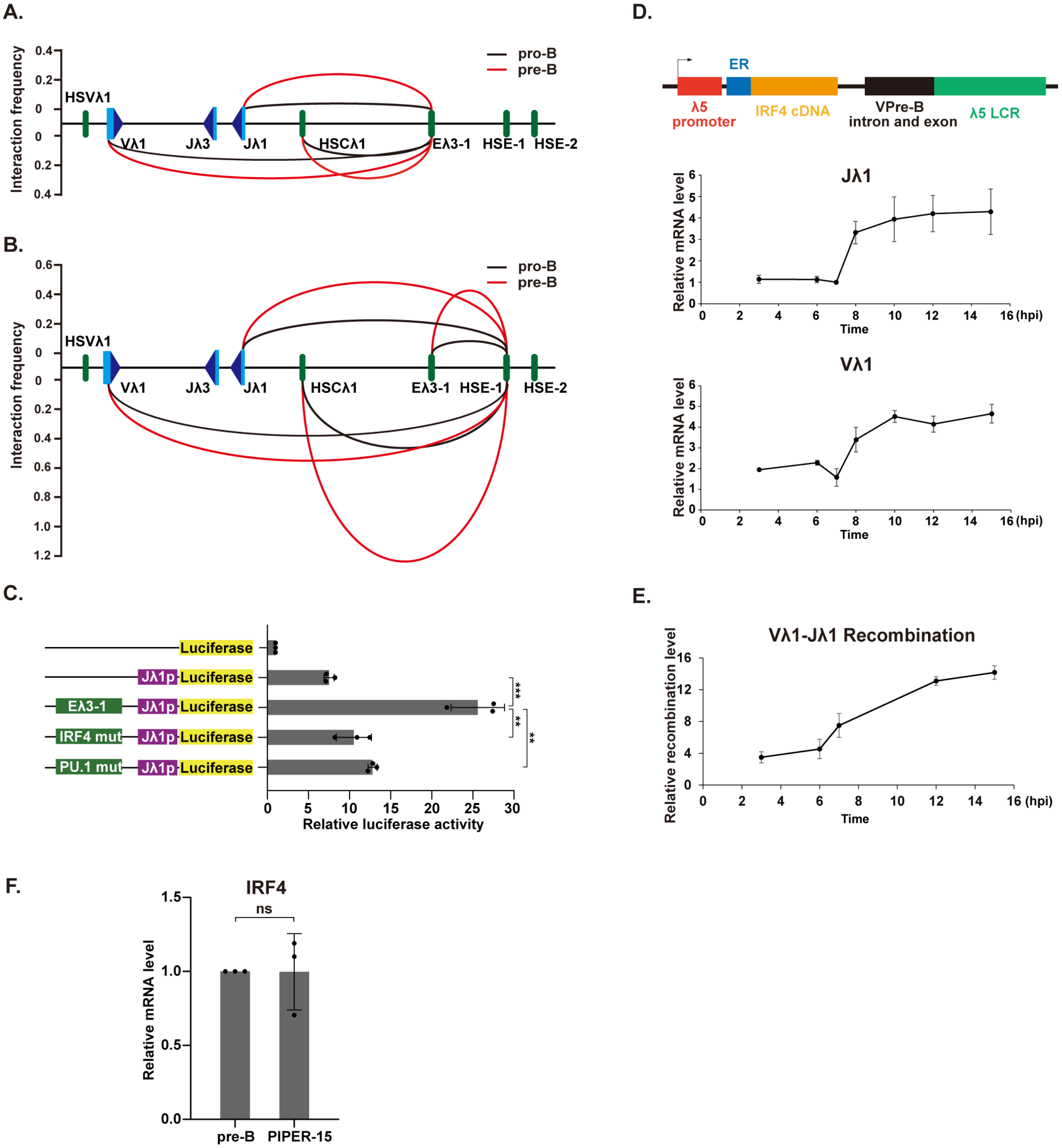
A.
Interaction frequency
pro-B
pre-B
HSVλ1
Vλ1
Jλ3
Jλ1
HSCλ1
Eλ3-1
HSE-1
HSE-2
B.
Interaction frequency
pro-B
pre-B
HSVλ1
Vλ1
Jλ3
Jλ1
HSCλ1
Eλ3-1
HSE-1
HSE-2
C.
Luciferase
Jλ1p
Eλ3-1
IRF4 mut
PU.1 mut
***
**
**
Relative luciferase activity
D.
ER
λ5 promoter
IRF4 cDNA
VPre-B intron and exon
λ5 LCR
Jλ1
Relative mRNA level
Time
(hpi)
Vλ1
Relative mRNA level
Time
(hpi)
E.
Vλ1-Jλ1 Recombination
Relative recombination level
Time
(hpi)
F.
IRF4
ns
Relative mRNA level
pre-B
PIPER-15

Figure S1. The Igλ locus is contracted during the pro-B to pre-B transition, leading to PU.1 and IRF4-mediated activation.

A. Analysis of the relative interaction frequency of Dpn II fragments from the Eλ3-1 viewpoint in pro- and pre-B cells. The height of curves between Eλ3-1 and other genomic fragments represents the average value of interaction frequency obtained from two (pro-B) or three (pre-B) experimental repeats (Supplementary Table 1). Data were normalized using an interaction within the *Ercc3* locus.
B. Analysis of the relative interaction frequency of Dpn II fragments from the HSE-1 viewpoint in pro- and pre-B cells. The height of curves between HSE-1 and other genomic fragments represents the average value of interaction frequency obtained from two (pro-B) or three (pre-B) experimental repeats (Supplementary Table 1). Data were normalized using an interaction within the *Ercc3* locus.
C. Luciferase activity driven by the wild type and mutant Eλ3-1 enhancers in 103/BCL-2 cells that had been temperature shifted to 39.5°C (24). The Jλ1 promoter increases luciferase activity by ~7-fold compared to the empty vector; Eλ3-1 gives a further 3-fold increase. To generate the PU.1mut and IRF4mut constructs, the core consensus of the PU.1 and IRF4 binding sites within Eλ3-1 were mutated from "GAAA" to "TCAA" and "GGAA" to "CCAA", respectively.
D. Upper: Schematic of the *Irf4-ER* cassette used to generate transgenic mice. Lower: Temporal analysis of Vλ1 and Jλ1 transcription by RT-qPCR in primary *Irf4-ER* pro-B cells following induction. Data are normalized to *Hprt* expression.
E. Temporal analysis of Vλ1-Jλ1 recombination by nested qPCR following induction of primary *Irf4-ER* pro-B cells. Samples were normalised using Intgene III.
F. *Irf4* expression in primary pre-B and PIPER-15 cells.
Error bars show standard error of the mean (SEM) from three biological replicates.

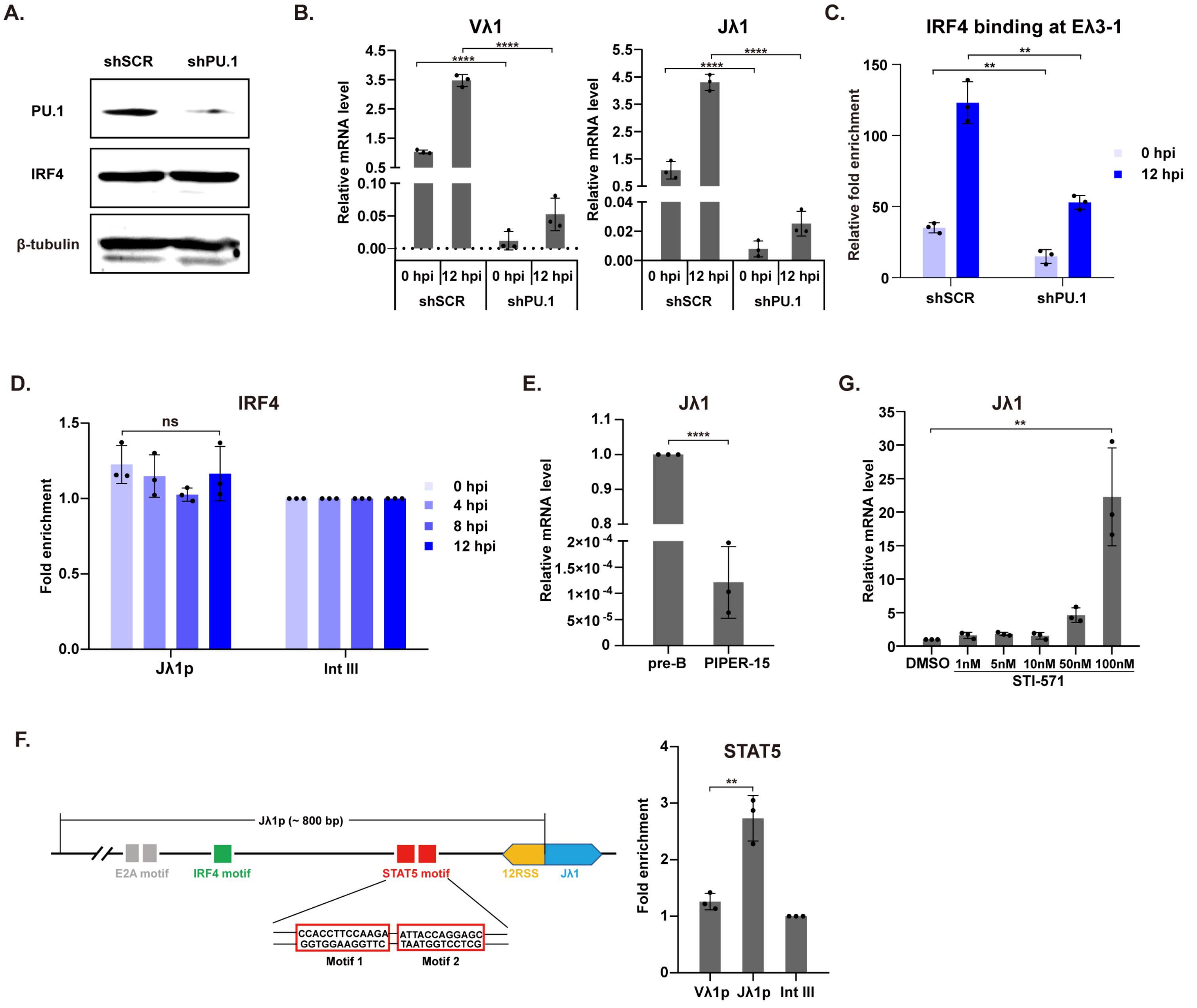

A.
shSCR
shPU.1
PU.1
IRF4
β-tubulin
B.
Vλ1
Relative mRNA level
****
****
0 hpi
12 hpi
shSCR
shPU.1
Jλ1
C.
IRF4 binding at Eλ3-1
Relative fold enrichment
**
0 hpi
12 hpi
D.
IRF4
Fold enrichment
ns
4 hpi
8 hpi
Jλ1p
Int III
E.
pre-B
PIPER-15
G.
DMSO
1nM
5nM
10nM
50nM
100nM
STI-571
F.
Jλ1p (~ 800 bp)
E2A motif
IRF4 motif
STAT5 motif
12RSS
CCACCTTCCAAGA
GGTGGAAGGTTC
ATTACCAGGAGC
TAATGGTCCTCG
Motif 1
Motif 2
STAT5
Vλ1p

Figure S2. The Jλ1 promoter is repressed in PIPER-15 cells

A. Western blot showing PU.1 expression levels in PIPER-15 cells expressing scrambled (shSCR) or shRNA targeting *Spi1* (shPU.1). β-tubulin was used as a loading control.
B. RT-qPCR analysis of Vλ1 and Jλ1 transcription in PIPER-15 cells expressing scrambled (shSCR; 0 and 12 hpi) or shRNA targeting *Spi1* (shPU.1; 0 and 12 hpi). The shSCR control values are highly reproducible; the 12 hpi shSCR data are from other knock-down experiments. These were normalised to 0 hpi shSCR data from this experiment and are shown here to easily compare the effects of the PU.1 knock-down.
C. IRF4 binding to Eλ3-1, analysed by ChIP-qPCR, in induced and uninduced PIPER-15 cells. The fold enrichment at Eλ3-1 and Intgene III is shown in cells expressing scrambled (shSCR), or shRNA against *Spi1* (shPU.1). All values are normalized to binding at Intgene III as a negative control.
D. IRF4 binding to Jλ1p, analysed by ChIP-qPCR, in PIPER-15 cells following induction. The fold enrichment at Jλ1p and Intgene III is shown. All values are normalized to binding at Intgene III as a negative control.
E. The level of Jλ1 transcription in pre-B and uninduced PIPER-15 cells. Data are normalized to *Hprt* expression.
F. Left: STAT5 binding motifs in the Jλ1 promoter, as predicted by the LASAGNA-search tool. Right: ChIP-qPCR confirmed the STAT5 enrichment at Jλ1p. All values are normalized to binding at Intgene III as a negative control.
G. Analysis of Jλ1 transcription by RT-qPCR in PIPER-15 cells with and without treatment with Imatinib. Data are normalized to *Hprt* expression.
Error bars show standard error of the mean (SEM) from three biological replicates.

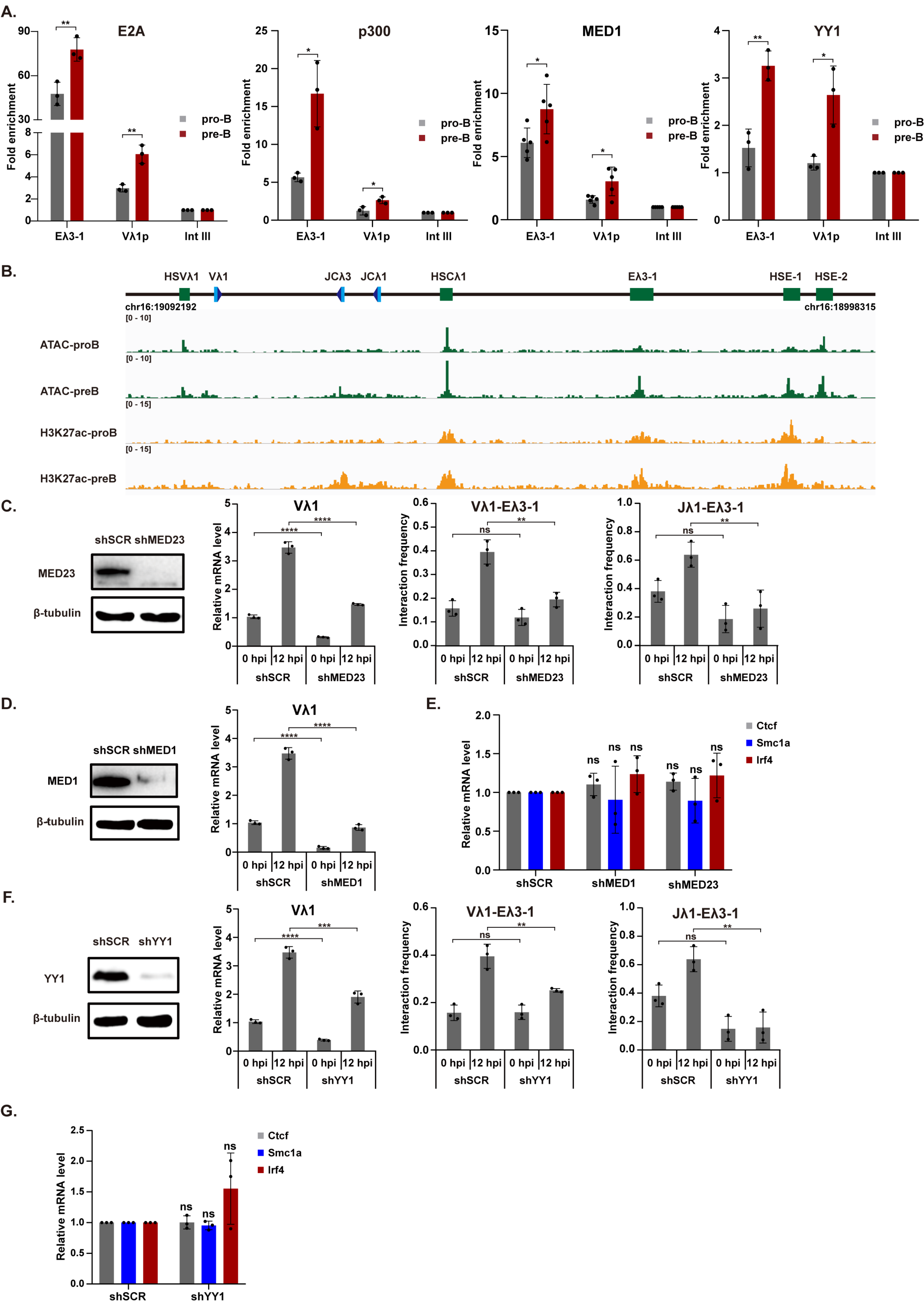
A.
E2A
p300
MED1
YY1
Fold enrichment
pro-B
pre-B
Eλ3-1
Vλ1p
Int III
B.
HSVλ1
Vλ1
JCλ3
JCλ1
HSCλ1
Eλ3-1
HSE-1
HSE-2
chr16:19092192
chr16:18998315
ATAC-proB
ATAC-preB
H3K27ac-proB
H3K27ac-preB
C.
shSCR shMED23
MED23
β-tubulin
Vλ1
Vλ1-Eλ3-1
Jλ1-Eλ3-1
Relative mRNA level
Interaction frequency
0 hpi
12 hpi
shSCR
shMED23
D.
shSCR shMED1
MED1
β-tubulin
shMED1
E.
Ctcf
Smc1a
Irf4
F.
shSCR
shYY1
YY1
G.

Figure S3. Multiple transcription factors establish Eλ3-1-Vλ1p contacts.

A. E2A, p300, MED1 and YY1 binding was analysed by ChIP-qPCR in primary mouse pro- and pre-B cells. The fold enrichment over input DNA at Eλ3-1, Vλ1p and Intgene III is shown. All values are normalized to binding at Intgene III as a negative control.
B. ATAC-seq and H3K27ac ChIP-seq from murine primary pro- and pre-B cells mapped to the 3' half of the Igλ locus. Increased accessibility, as detected by ATAC-seq is clearly visible between pro- and pre-B cells. The increase in H3K27Ac is smaller; this may be because the primary cells were purified by flow cytometry in the absence of deacetylase inhibitors (Koohy et al., (2018) *Genome Biol.,* **19**, 126) which may have resulted in some loss of H3K27Ac.
C. Left: Western blot showing MED23 expression levels in PIPER-15 cells expressing scrambled (shSCR) or shRNA targeting *Med23* (shMED23). β-tubulin was used as a loading control. Middle: RT-qPCR analysis of Vλ1 transcription in MED23 knock-down cells. Right: 3C analysis of the interaction frequency between Eλ3-1 and Vλ1 as well as Jλ1 in shMED23 PIPER-15 cells.
D. Left: Western blot showing MED1 expression levels in PIPER-15 cells expressing scrambled (shSCR) or shRNA targeting *Med1* (shMED1). β-tubulin was used as a loading control. Right: RT-qPCR analysis of Vλ1 transcription in MED1 knock-down cells
E. RT-qPCR analysis of *Ctcf*, *Smc1a* and *Irf4* expression in PIPER-15 cells expressing scrambled (shSCR) or shRNA targeting *Med1* (shMED1) or *Med23* (shMED23). Data are normalized to *Hprt* expression.
F. Left: Western blot showing YY1 expression levels in in PIPER-15 cells expressing scrambled (shSCR) or shRNA targeting *Yy1* (shYY1). β-tubulin was used as a loading control. Middle: RT-qPCR analysis of Vλ1 transcription in YY1 knock-down cells. Right: 3C analysis of the interaction frequency between Eλ3-1 and Vλ1 as well as Jλ1 in PIPER-15 cells expressing scrambled (shSCR) or shRNA targeting *Yy1* (shYY1).
G. RT-qPCR analysis of *Ctcf*, *Smc1a* and *Irf4* expression in PIPER-15 cells expressing scrambled (shSCR) or shRNA targeting *Yy1* (shYY1). Data are normalized to *Hprt* expression. Error bars show standard error of the mean (SEM) from three biological replicates.

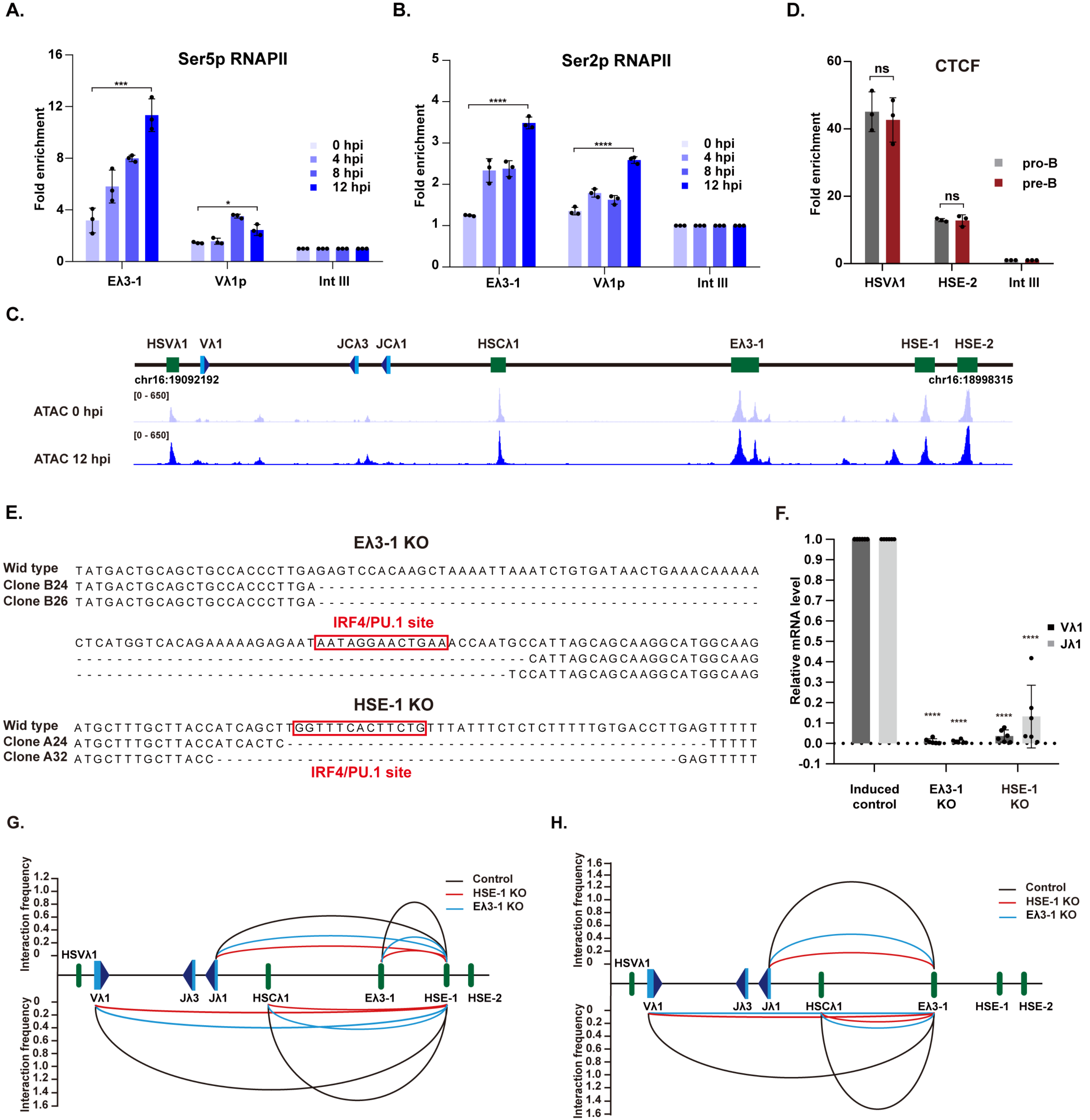
A.
Ser5p RNAPII
Fold enrichment
0 hpi
4 hpi
8 hpi
12 hpi
Eλ3-1
Vλ1p
Int III
B.
Ser2p RNAPII
Fold enrichment
0 hpi
4 hpi
8 hpi
12 hpi
Eλ3-1
Vλ1p
Int III
D.
CTCF
Fold enrichment
pro-B
pre-B
ns
HSVλ1
HSE-2
Int III
C.
HSVλ1
Vλ1
JCλ3
JCλ1
HSCλ1
Eλ3-1
HSE-1
HSE-2
chr16:19092192
chr16:18998315
[0 - 650]
ATAC 0 hpi
ATAC 12 hpi
E.
Eλ3-1 KO
Wid type TATGACTGCAGCTGCCACCCTTGAGAGTCCACAAGCTAAAATTAAATCTGTGATAACTGAAACAAAAA
Clone B24 TATGACTGCAGCTGCCACCCTTGA
Clone B26 TATGACTGCAGCTGCCACCCTTGA
IRF4/PU.1 site
CTCATGGTCACAGAAAAAGAGAATAATAGGAACTGAAACCAATGCCATTAGCAGCAAGGCATGGCAAG
CATTAGCAGCAAGGCATGGCAAG
TCCATTAGCAGCAAGGCATGGCAAG
HSE-1 KO
Wid type ATGCTTTGCTTACCATCAGCTTGGTTTCACTTCTGTTTATTTCTCTCTTTTTGTGACCTTGAGTTTTT
Clone A24 ATGCTTTGCTTACCATCACTC TTTTT
Clone A32 ATGCTTTGCTTACC GAGTTTTT
IRF4/PU.1 site
F.
Relative mRNA level
Vλ1
Jλ1
Induced control
Eλ3-1 KO
HSE-1 KO
G.
Interaction frequency
Control
HSE-1 KO
Eλ3-1 KO
HSVλ1
Vλ1
Jλ3
Jλ1
HSCλ1
Eλ3-1
HSE-1
HSE-2
H.
Interaction frequency
Control
HSE-1 KO
Eλ3-1 KO
HSVλ1
Vλ1
Jλ3
Jλ1
HSCλ1
Eλ3-1
HSE-1
HSE-2

Figure S4. IRF4 triggers the formation of an enhancer hub and RNAPII recruitment during the pro-B to pre-B transition

A-B. Binding of RNAPII, phosphorylated at (A) serine-5 or (B) serine-2 of the C-terminal repeat domain, was analysed by ChIP-qPCR in PIPER-15 cells following induction. The fold enrichment over input DNA at Eλ3-1, Vλ1p and Intgene III (negative control region) is shown. All values are normalized to binding at Intgene III as a negative control.
C. ATAC-seq from uninduced (0 hpi) and induced (12 hpi) PIPER-15 cells, mapped to the 3' half of the Igλ locus (chr 16:19092192-18998315; mm9).
D. CTCF binding to HSE-2 and HSVλ1 in primary pro-B and pre-B cells. The fold enrichment at HSE-2, HSVλ1 and Intgene III is shown. All values are normalized to binding at Intgene III as a negative control.
E. Sequence alignments of clones bearing deletions of the PU.1/IRF4 binding site at HSE-1 (upper) or Eλ3-1 (lower). Two clones are shown per enhancer. Analysis of the deleted sequence shows that the IRF4/PU.1 site is removed from each enhancer but binding sites for the other factors we discuss, remain intact. A summary of motifs within the deleted region, detected by Find Individual Motif Occurrences (FIMO), and the relative expression of the corresponding transcription factors in early B cells, are shown in Supplementary Table 2.
F. The level of Vλ1 and Jλ1 transcription analyzed by RT-qPCR in induced PIPER-15 cells where HSE-1 or Eλ3-1 had been knocked out. Data are normalized to *Hprt* expression.
G. Analysis of the relative interaction frequency of Dpn II fragments from the HSE-1 viewpoint in induced Eλ3-1 and HSE-1 knockout PIPER-15 cells. The height of curves between HSE-1 and other genomic fragments represents the average value of interaction frequency obtained from two experimental repeats (Supplementary Table 1). Data were normalized using an interaction within the *Ercc3* locus.
H. Analysis of the relative interaction frequency of Dpn II fragments from the Eλ3-1 viewpoint in induced Eλ3-1 and HSE-1 knockout PIPER-15 cells. The height of curves between Eλ3-1 and other genomic fragments represents the average value of interaction frequency obtained from two experimental repeats (Supplementary Table 1). Data were normalized using an interaction within the *Ercc3* locus.
Error bars show standard error of the mean (SEM) from three biological replicates.

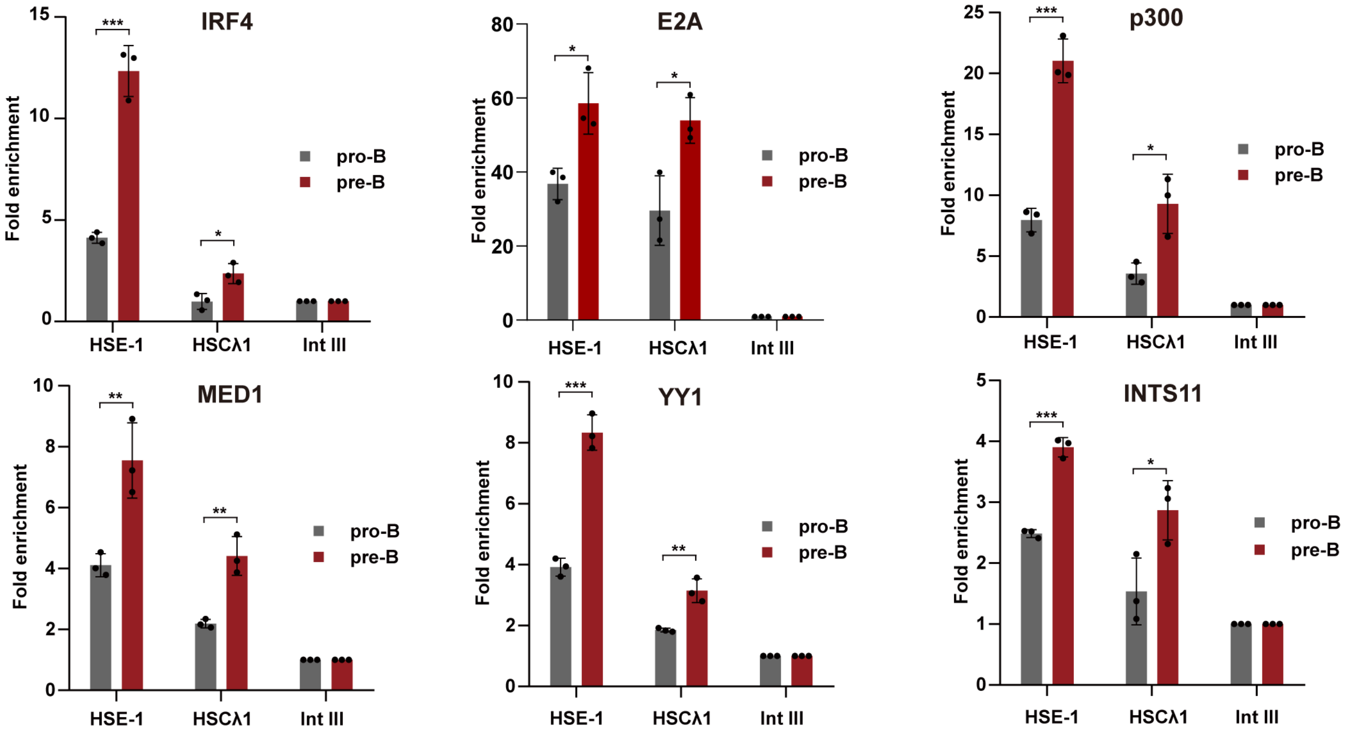
IRF4
Fold enrichment
0
5
10
15
***
*
pro-B
pre-B
HSE-1
HSCλ1
Int III
E2A
Fold enrichment
0
20
40
60
80
*
*
pro-B
pre-B
HSE-1
HSCλ1
Int III
p300
Fold enrichment
0
5
10
15
20
25
***
*
pro-B
pre-B
HSE-1
HSCλ1
Int III
MED1
Fold enrichment
0
2
4
6
8
10
**
**
pro-B
pre-B
HSE-1
HSCλ1
Int III
YY1
Fold enrichment
0
2
4
6
8
10
***
**
pro-B
pre-B
HSE-1
HSCλ1
Int III
INTS11
Fold enrichment
0
1
2
3
4
5
***
*
pro-B
pre-B
HSE-1
HSCλ1
Int III

Figure S5. Transcription factor recruitment to HSE.1 and HSCλ1

IRF4, E2A, p300, MED1, YY1 and INTS11 binding was analysed by ChIP-qPCR in primary mouse pro-B and pre-B cells. The fold enrichment over input DNA at HSCλ1, HSE-1 and Intgene III is shown. All values are normalized to binding at Intgene III as a negative control. Error bars show standard error of the mean (SEM) from three biological replicates.

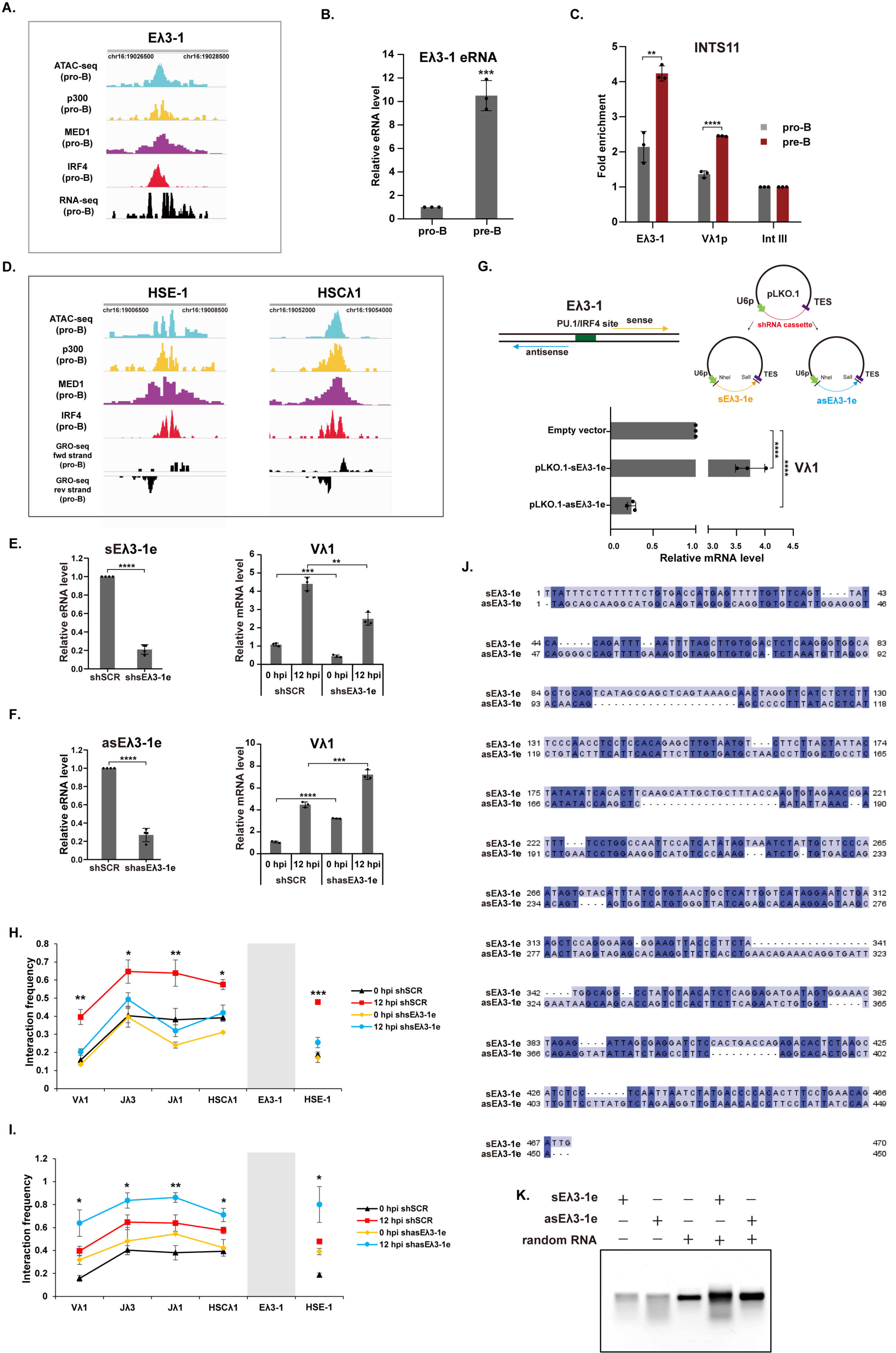
A.
Eλ3-1
chr16:19026500
chr16:19028500
ATAC-seq (pro-B)
p300 (pro-B)
MED1 (pro-B)
IRF4 (pro-B)
RNA-seq (pro-B)
B.
Eλ3-1 eRNA
Relative eRNA level
***
pro-B
pre-B
C.
INTS11
Fold enrichment
**
****
pro-B
pre-B
Eλ3-1
Vλ1p
Int III
D.
HSE-1
HSCλ1
chr16:19006500
chr16:19008500
chr16:19052000
chr16:19054000
ATAC-seq (pro-B)
p300 (pro-B)
MED1 (pro-B)
IRF4 (pro-B)
GRO-seq fwd strand (pro-B)
GRO-seq rev strand (pro-B)
E.
sEλ3-1e
Relative eRNA level
****
shSCR
shsEλ3-1e
Vλ1
Relative mRNA level
***
**
0 hpi
12 hpi
shSCR
shsEλ3-1e
F.
asEλ3-1e
Relative eRNA level
****
shSCR
shasEλ3-1e
Vλ1
Relative mRNA level
****
***
0 hpi
12 hpi
shSCR
shasEλ3-1e
G.
Eλ3-1
PU.1/IRF4 site
sense
antisense
pLKO.1
U6p
TES
shRNA cassette
NheI
SalI
sEλ3-1e
asEλ3-1e
Empty vector
pLKO.1-sEλ3-1e
pLKO.1-asEλ3-1e
****
****
Vλ1
Relative mRNA level
H.
Interaction frequency
0 hpi shSCR
12 hpi shSCR
0 hpi shsEλ3-1e
12 hpi shsEλ3-1e
Vλ1
Jλ3
Jλ1
HSCλ1
Eλ3-1
HSE-1
I.
Interaction frequency
0 hpi shSCR
12 hpi shSCR
0 hpi shasEλ3-1e
12 hpi shasEλ3-1e
Vλ1
Jλ3
Jλ1
HSCλ1
Eλ3-1
HSE-1
J.
sEλ3-1e
asEλ3-1e
K.
sEλ3-1e
asEλ3-1e
random RNA

Figure S6. The Eλ3-1 enhancer produces bidirectional enhancer RNAs during Igλ activation

A. RNA-seq data from pro-B cells was re-analyzed using the Galaxy server. Signal peaks of ATAC-seq and ChIP-seq data from pro-B cells map to the central region of the Eλ3-1 enhancer. Visualization of the mapped reads was performed using IGV. Genomic coordinates of Eλ3-1 are shown.
B. Transcription of the Eλ3-1 enhancer in primary pro-B and pre-B cells, determined by qPCR. Data are normalized to *Hprt* expression.
C. Integrator binding at the Eλ3-1 enhancer and Vλ1 promoter as determined by ChIP-qPCR in primary pro-B and pre-B cells. The fold enrichment at Eλ3-1, Vλ1p and Intgene III is shown. All values are normalized to binding at Intgene III as a negative control.
D. GRO-seq data from pro-B cells was re-analyzed using the Galaxy server. Signal peaks of ATAC-seq and ChIP-seq data from pro-B cells map to the central region of the HSCλ1 as well as HSE-1. Visualization of the mapped reads was performed using IGV. Genomic coordinates of HSE-1 and HSCλ1 are shown.
E. RT-qPCR analysis of sense Eλ3-1 eRNA (sEλ3-1e) and Vλ1 transcription in PIPER-15 cells expressing scrambled shRNA (shSCR) or shRNA targeting sense Eλ3-1 eRNA (shsEλ3-1e). Data are normalized to expression of the housekeeping gene, *Hprt*.
F. RT-qPCR analysis of antisense Eλ3-1 eRNA (asEλ3-1e) and Vλ1 transcription in PIPER-15 cells expressing scrambled shRNA (shSCR) or shRNA targeting antisense Eλ3-1 eRNA (shasEλ3-1e). Data are normalized to expression of the housekeeping gene, *Hprt*.
G. The lentiviral vector, pLKO.1, was modified to express eRNAs. Vλ1 transcription was analysed following over-expression of sense (sEλ3-1e) or antisense (asEλ3-1e) Eλ3-1 eRNA in PIPER-15 cells. Data are normalized to *Hprt* expression.
H-I. Analysis of the relative interaction frequency of Dpn II fragments from the Eλ3-1 enhancer in PIPER-15 cells expressing scrambled (shSCR), or shRNAs against (H) sense (shsEλ3-1e) or (I) antisense (shasEλ3-1e) eRNAs. The plots show the significance of the difference in interactions at 12 hpi between shSCR and (H) shsEl3-1e or (I) shasEλ3-1e.
J. Sequence alignment of sense (sEλ3-1e) and antisense (asEλ3-1e) Eλ3-1 eRNAs.
K. Native agarose gel electrophoresis of sense (sEλ3-1e) and antisense (asEλ3-1e) Eλ3-1 eRNAs with and without hybridisation to a random RNA.
Error bars show standard error of the mean (SEM) from three biological replicates.
