# Supplementary material for: Temporal analyses reveal a pivotal role for sense and antisense enhancer RNAs in coordinate immunoglobulin lambda locus activation
Source: Nucleic Acids Res. 2023 Sep 13;51(19):10344–63. doi: 10.1093/nar/gkad741 (PMC10602925; doi:10.1093/nar/gkad741)
Supplement: gkad741_Supplemental_Files [file gkad741_supplemental_files.zip › Legends to Supplementary Tables.docx]

**Legends to Supplementary Tables**

**Supplementary Table 1**

Table showing reagents/datasets used in this study. Tab 1: Oligonucleotides used; Tab 2: Antibodies used; Tab 3: Next Generation Sequencing (NGS) datasets used; Tab 4: Repeats of the 3C data from which the plots in Figures 4C, 5, 6E and Supplementary Figures S1A, S1B, S4G, S4H, S6H and S6I were derived.

**Supplementary Table 2**

Tables showing the consensus transcription factor binding sites in Eλ3-1 (Tab 1) and HSE-1 (Tab 2) as determined by FIMO (Find Individual Motif Occurrences). A cut-off q value of <0.01 was used. FPKM (Fragments Per Kilobase of transcript per Million mapped reads) values are shown from pre-B cell RNA-seq data.
